# Autophagy-induced senescence is regulated by p38α signaling

**DOI:** 10.1038/s41419-019-1607-0

**Published:** 2019-05-15

**Authors:** Konstantin Slobodnyuk, Nevenka Radic, Saška Ivanova, Anna Llado, Natalia Trempolec, Antonio Zorzano, Angel R. Nebreda

**Affiliations:** 1 Institute for Research in Biomedicine (IRB Barcelona), The Barcelona Institute of Science and Technology, 08028 Barcelona, Spain; 2 Department of Biochemistry and Molecular Biomedicine, University of Barcelona, Faculty of Biology, 08028 Barcelona, Spain; 3 CIBER de Diabetes y Enfermedades Metabólicas Asociadas (CIBERDEM), Instituto de Salud Carlos III, Barcelona, Spain; 4 ICREA, Pg. Lluís Companys 23, 08010 Barcelona, Spain

**Keywords:** Kinases, Macroautophagy

## Abstract

Apoptosis and senescence are two mutually exclusive cell fate programs that can be activated by stress. The factors that instruct cells to enter into senescence or apoptosis are not fully understood, but both programs can be regulated by the stress kinase p38α. Using an inducible system that specifically activates this pathway, we show that sustained p38α activation suffices to trigger massive autophagosome formation and to enhance the basal autophagic flux. This requires the concurrent effect of increased mitochondrial reactive oxygen species production and the phosphorylation of the ULK1 kinase on Ser-555 by p38α. Moreover, we demonstrate that macroautophagy induction by p38α signaling determines that cancer cells preferentially enter senescence instead of undergoing apoptosis. In agreement with these results, we present evidence that the induction of autophagy by p38α protects cancer cells from chemotherapy-induced apoptosis by promoting senescence. Our results identify a new mechanism of p38α-regulated basal autophagy that controls the fate of cancer cells in response to stress.

## Introduction

Cells exposed to a variety of stress signals can undergo a process of programmed cell death known as apoptosis, or can enter into an irreversible cell cycle arrest called senescence^1–3^. These two processes are closely related cellular failsafe programs, which are associated with the response of cancer cells to chemotherapeutic drugs such as doxorubicin^4–6^. A similar dichotomy between entering senescence or apoptosis has been reported in melanoma cancer cells exposed to a BRAF inhibitor^7^ and in cervical cancer cell lines treated with cisplatin^8^. Overall, the choice between senescence and apoptosis seems to be determined by both the type of cancer cell and the stress stimuli.

Autophagy is an important homeostatic mechanism of lysosome-based degradation of cytosolic constituents, which is often activated in response to stress and has been linked to both apoptosis and senescence^9,10^. Generally, autophagy interferes with apoptosis induction in cancer cells^11^, although, in particular cases autophagy has been also described to lead to cell death^12^. Likewise, autophagy can function as a pro-senescent mechanism in cancer cells^13^, but impaired autophagy can trigger senescence in muscle stem cells^14^ or breast cancer cells^15^. These results indicate that the outcome of autophagy is highly dependent on the cell type and context.

Cell fate decisions are often influenced by the treatment strength (i.e., drug concentration), but we know very little on the factors that determine whether cells undergo apoptosis or senescence. Noteworthy, both programs have been linked to the activation of the stress-activated kinase p38α. This pathway is widely used by eukaryotic cells to interpret extracellular stimuli, in particular stress signals^16^. Once activated, p38α can phosphorylate diverse substrates, which subsequently regulate a variety of cellular functions^17^. There is evidence linking p38α activation to the induction of both apoptosis and premature senescence^16,18–20^. In addition, p38α activity has been implicated in the regulation of stress-induced autophagy. Most reports document that p38α inhibits autophagy, for example in lipopolysaccharide (LPS)-treated primary microglia cells^21^ and LPS-treated fibroblasts^22^, or in starved HEK293 cells^23^. However, there is a report showing that autophagy induced by amino-acid starvation in cancer cells is mediated by MK2 and MK3, two kinases downstream of p38α^24^ Of note, several studies on the role of p38α in autophagy are based on the use of pyridinyl imidazole compounds such as SB203580, which can modulate the autophagic flux independently of p38α inhibition^25^. Therefore, the implication of p38α in autophagy should be re-evaluated using appropriate reagents.

In this study, we show that p38α activation suffices to strongly enhance the basal levels of autophagy. We also provide evidence that p38α-induced autophagy regulates the fate of cancer cells treated with chemotherapeutic drugs.

## Results

### p38α activation suffices to induce autophagy

**Fig. 1:**
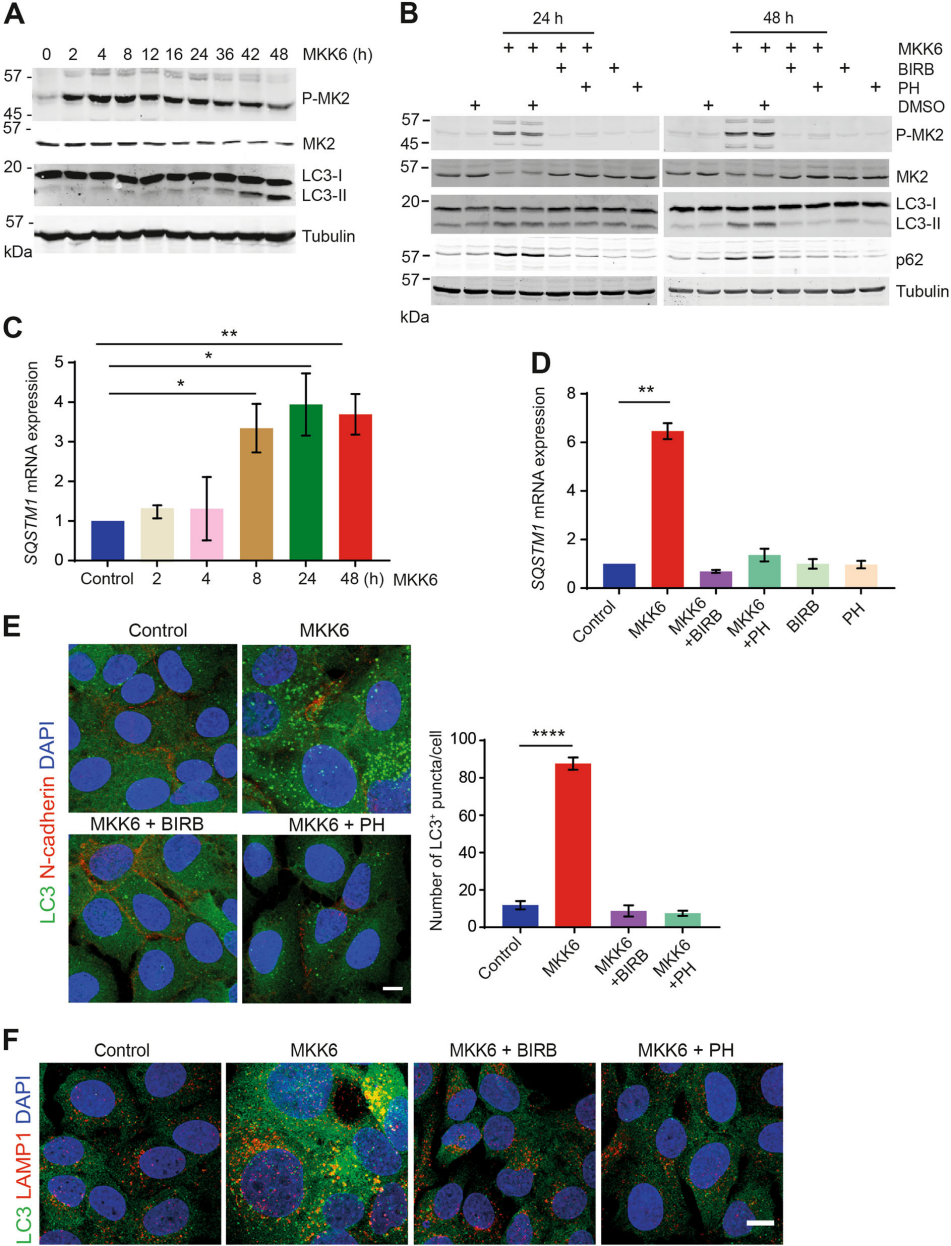
Activation of p38α suffices to induce autophagy. U2OS cells expressing a Tet-regulated construct were either mock treated (control) or treated with tetracycline for the indicated times to induce the expression of constitutively active MKK6. **a** Total cell lysates were analyzed by immunoblotting using the indicated antibodies. **b** Control and MKK6-expressing cells were treated with the p38α inhibitors PH797804 (PH) and BIRB796 (BIRB), or with DMSO for the indicated times, and total cell lysates were analyzed by immunoblotting. **c**, **d** Control and MKK6-expressing cells were grown in the presence or absence of the p38α inhibitors PH or BIRB for the indicated times (**c**) or for 48 h (**d**) and the levels of *SQSTM1* mRNA encoding p62 were analyzed by qRT-PCR. Results are presented as fold change towards the control. **e** Immunofluorescence detection of LC3^+^ puncta (autophagosomes) in U2OS cells expressing MKK6 for 48 h in the presence or absence of PH or BIRB. The histogram shows the quantification of puncta. Bar = 10 μm. **f** Representative immunofluorescence images to illustrate the colocalization of LC3^+^ autophagosomes (green) and LAMP1^+^ lysosomes (red) at 48 h after MKK6 induction, either alone or together with PH or BIRB. Bar = 10 μm. Differences between control and MKK6-expressing cells were analyzed using the unpaired Student's *t* test, (****) *p* < 0.00001, (**) *p* < 0.001, (*) *p* < 0.01

We have generated an inducible system in U2OS cells that triggers the activation of p38α signaling by expression of constitutively active MKK6, a p38 MAPK-specific MAP2K, thus allowing us to study how this pathway regulates cellular processes in the absence of other stress-activated pathways^26^. We used this system to investigate the effect of p38α activation on basal macroautophagy. Immunoblotting analysis revealed that the activation of p38α, as monitored by the phosphorylation of its substrate MK2, led to enhanced conversion of LC3-I into the lipidated form LC3-II, which started to be detected at 24–36 h after p38α activation and showed a clear accumulation at 42–48 h (Fig. 1a). The MKK6-induced changes in LC3-II levels were reversed by incubation with the chemical inhibitors PH797804 or BIRB796, indicating that they were triggered by p38α activation (Fig. 1b). We also found that MKK6 expression induced the accumulation of p62 protein, which is considered a marker of autophagy^27^, but this was not observed in the presence of p38α inhibitors (Fig. 1b). It has been reported that p38α can induce the expression of the *SQSTM1* gene encoding p62^28^, and we observed *SQSTM1* mRNA upregulation starting at 8 h after MKK6 induction, which was maintained until 48 h (Fig. 1c). Inhibition of p38α decreased the level of *SQSTM1* mRNA in MKK6-expressing cells to the levels of control cells (Fig. 1d). The ability of p38α to induce *SQSTM1* mRNA upregulation suggests that p62 protein levels are not a reliable marker to study autophagy regulation when p38α is involved.

To confirm the enhanced LC3 lipidation detected by immunoblotting, we analyzed the endogenous LC3 levels by immunofluorescence. We found a significantly upregulated number of LC3^+^ puncta (autophagosomes) at 48 h after MKK6 induction, but this was not observed in the presence of p38α inhibitors (Fig. 1e). Moreover, 48 h after MKK6 induction, we observed the colocalization of endogenous LC3 protein with the lysosome marker LAMP1, which was impaired by p38α inhibitors (Fig. 1f), suggesting that p38α induces the fusion of autophagosomes with lysosomes for further processing.

MKK6 expression in U2OS cells leads to the activation of both p38α and p38γ^26^. The ability of PH797804 to inhibit MKK6-induced autophagosome formation suggested that this was mediated by p38α. We genetically ablated p38α or p38γ using the CRISPR/Cas9 system (Supplementary Fig. 1A), and did not observe significant changes in the p38α expression levels upon p38γ deletion or vice versa (Supplementary Fig. 1B). We confirmed that deletion of p38α but not p38γ impaired the MKK6-induced p62 upregulation (Supplementary Fig. 1A). However, immunoblotting analysis showed some reduction in the MKK6-induced LC3-II levels upon p38γ deletion but to a much lower extent than upon p38α deletion (Supplementary Fig. 1A). We confirmed by immunofluorescence that p38α deletion ablated the MKK6-induced increase in LC3^+^ puncta, but the number of LC3^+^ puncta detected in MKK6-expressing p38γ KO cells was also reduced in comparison with MKK6-expressing wild type (WT) cells. PH797804 or BIRB796 restored the number of autophagosomes in MKK6-expressing p38γ KO cells to the levels of untreated WT cells, supporting a key role for p38α (Supplementary Fig. 1C and D). Moreover, MKK6 expression not only increased the number of autophagosomes but also their average size, which was mostly dependent on p38α (Supplementary Fig. 1D). Collectively, these results indicate that p38α activation suffices to increase the number and size of autophagosomes, although p38γ might also have a minor contribution to autophagy induction in MKK6-expressing cells.

### p38α activation increases the autophagic flux

**Fig. 2:**
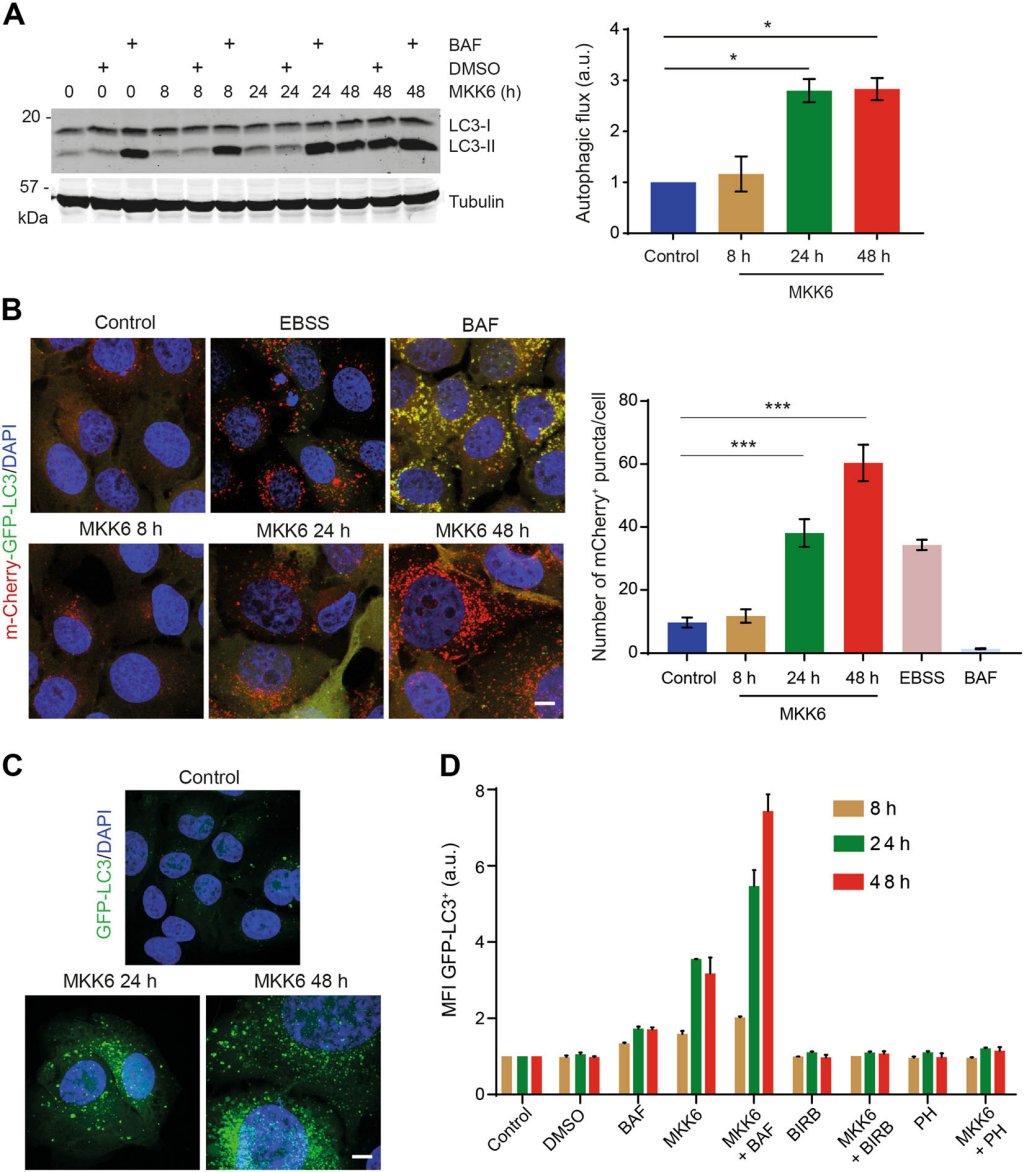
p38α activation increases the autophagic flux. U2OS cells expressing a Tet-regulated construct were either mock treated (control) or treated with tetracycline for the indicated times to induce the expression of constitutively active MKK6. **a** Cells were treated with the autophagy inhibitor bafilomycin A1 (BAF) for 4 h at the indicated times after MKK6 induction, and then were lysed and analyzed by immunoblotting using the indicated antibodies. The densitometric quantification of the ratio of the lipidated LC3 (LC3-II) band to tubulin in the immunoblotting was performed. The autophagic flux was calculated by subtraction of the ratio LC3-II/tubulin from cells that were treated or not treated with BAF for 4 h at the indicated times after MKK6 induction. **b** Immunofluorescence analysis of cells expressing mCherry-GFP-LC3 reporter at the indicated times after MKK6 induction. The number of red dots increased in cells exposed to autophagy-inducing EBSS media for 8 h, and decreased by treatment with BAF for 4 h. The autophagic flux was determined by quantification of the number of red dots, performed by subtracting the number of green puncta from red puncta. Bar = 10 μm. **c** Detection of GFP-LC3^+^ autophagosomes at the indicated times after MKK6 induction in cells stably expressing GFP-LC3. **d** Mean fluorescence intensity (MFI) in arbitrary units (a.u.) was measured in GFP-LC3-expressing cells at the indicated times upon MKK6 induction in the presence or absence of the p38α inhibitors PH797804 (PH) or BIRB796 (BIRB). When indicated, cells were treated with BAF for 4 h. Differences between control and MKK6-expressing cells were analyzed using the unpaired Student's *t* test, (***) *p* < 0.0001, (*) *p* < 0.01

Autophagy induction correlates with the degradation of p62 and LC3-II proteins as well as the cargo inside the autophagosomes^29^. Nevertheless, an increased conversion of LC3-I to LC3-II detected by immunoblotting or an increased number of LC3^+^ puncta could reflect autophagy induction and thus increased autophagic degradation activity (autophagic flux increased). Alternatively, these changes could be owing to the inhibition of autophagolysosome degradation, resulting from either reduced fusion of autophagosomes with lysosomes or impaired autolysosomes digestion (autophagic flux inhibited). To evaluate the role of p38α in autophagic flux regulation, we combined MKK6 induction for different times with bafilomycin A1 treatment for 4 h. Quantification of LC3-II by immunoblotting showed increased autophagic flux starting at 24 h after MKK6 induction (Fig. 2a). To confirm these findings, we used a mCherry-green fluorescent protein (GFP)-LC3 reporter^30^. Immunofluorescence analysis showed an increased number of red LC3 puncta (mature autolysosomes), indicating increased autophagic flux, in cells expressing MKK6 for 24–48 h as well as in cells starved in Earle’s Balanced Salt Solution (EBSS) media (Fig. 2b). Importantly, the level of p38α-induced autophagic flux was comparable to that observed in starved cells. On the other hand, bafilomycin A1 caused the accumulation of yellow LC3 puncta while decreasing the number of red puncta, as expected from the autophagic flux blockage (Fig. 2b).

Further support for autophagic flux enhancement by p38α was obtained in cells expressing GFP-LC3. Immunofluorescence analysis revealed the accumulation of GFP-LC3^+^ puncta starting 24 h after p38α activation (Fig. 2c). The results were confirmed by measuring mean fluorescence intensity (MFI) by fluorescence-activated cell sorting (FACS)^14^ in GFP-LC3-expressing cells (Fig. 2d). These data indicate that p38α activation induces autophagosome formation with subsequent increase of the autophagic flux.

To investigate whether the observed changes in autophagy require continuous p38α activation, we added p38α inhibitors at different times after MKK6 induction (Supplementary Fig. 2A). We found that p38α inhibition 24 h after MKK6 induction restored the morphology observed in untreated cells (Supplementary Fig. 2B). Biochemical analysis confirmed that sustained p38α activity was required for both increased p62 expression and LC3 lipidation (Supplementary Fig. 2C). Furthermore, immunofluorescence analysis showed decreased number of autophagosomes upon treatment for 48 h with p38α inhibitors, either 24 h or 48 h after MKK6 induction (Supplementary Fig. 2D and E). These results indicate that the observed changes in autophagy require sustained p38α activation.

Next, we generated the same inducible system in Saos-2 cells, and found that MKK6 expression led to p38α activation (Supplementary Fig. 3A). Immunoblot analysis showed enhanced LC3-II levels starting 48 h after MKK6 induction (Supplementary Fig. 3B), suggesting autophagy induction. Immunofluorescence confirmed the p38α-induced accumulation of LC3^+^ puncta (Supplementary Fig. 3C). We also detected increased autophagic flux using the mCherry-GFP-LC3 reporter in MKK6-expressing cells (Supplementary Fig. 3D). These results demonstrate that p38α activation suffices to increase the autophagic flux in different cell lines.

### Autophagy induction by p38α through ULK1 phosphorylation

Mammalian autophagy induction is mediated mainly by two complexes: one contains the protein kinase ULK1, and the other one the class III phosphatidylinositol 3-kinase PIK3C3 with the regulatory subunits Beclin-1 and ATG14L, and the scaffold protein PIK3R4 (or Vps15)^31^. We investigated whether p38α could directly phosphorylate ULK1, which in turn induces autophagy by phosphorylating Beclin-1.

**Fig. 3:**
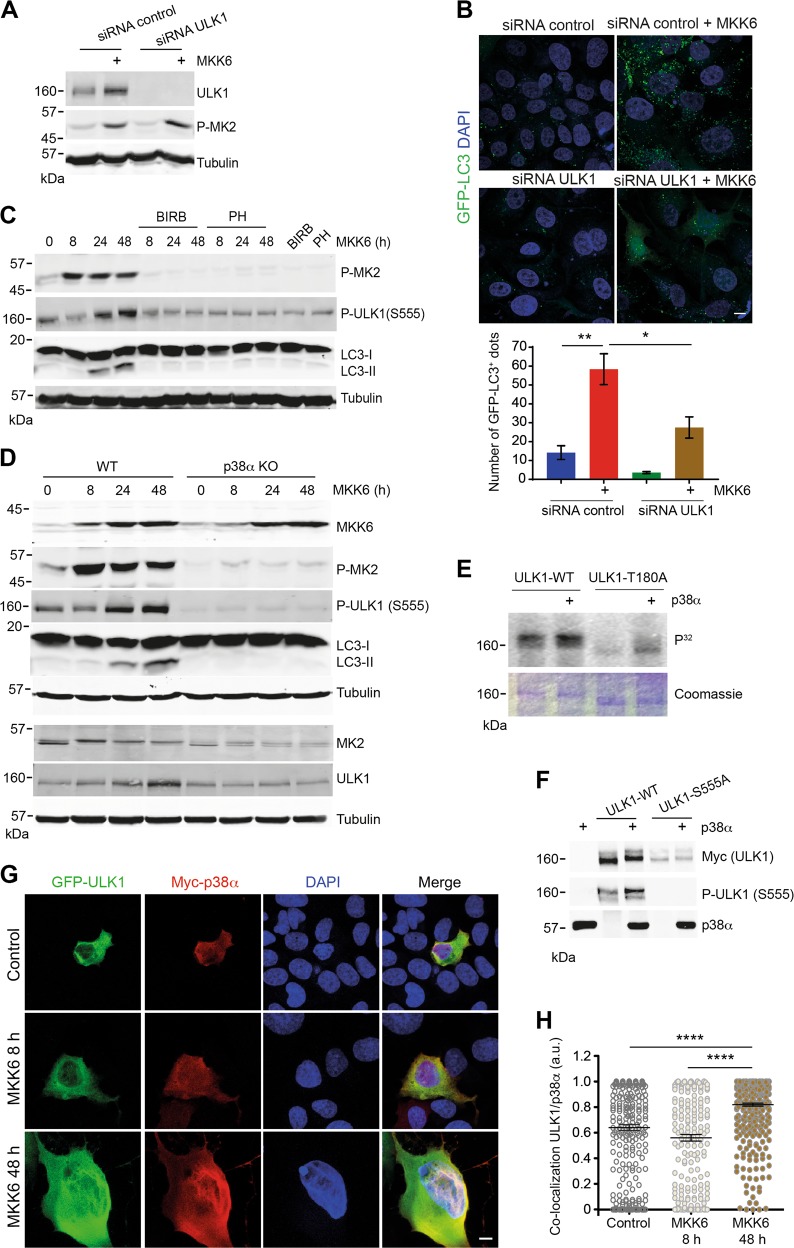
p38α-induced autophagy involves ULK1 phosphorylation. U2OS cells expressing a Tet-regulated construct were either mock treated (control) or treated with tetracycline for the indicated times to induce the expression of constitutively active MKK6. **a** Cells were treated with mock (control) or ULK1 siRNAs and then MKK6 was induced for 48 h. Cell lysates were analyzed by immunoblotting using the indicated antibodies. **b** Immunofluorescence analysis of GFP-LC3^+^ puncta in cells expressing MKK6 for 48 h and treated with control or ULK1 siRNA. The histogram shows the quantification of the GFP-LC3^+^ puncta. Bar = 10 μm. **c** Total lysates of MKK6-expressing cells incubated in the presence or absence of the p38α inhibitors PH797804 (PH) or BIRB796 (BIRB) were analyzed by immunoblotting. **d** Total lysates of cells wild type (WT) or knockout for p38α (p38α ΚΟ) expressing MKK6 for the indicated times were analyzed by immunoblotting. **e** Myc-ULK1 proteins either WT or the T180A mutant were overexpressed in HEK293 cells, immunoprecipitated and incubated with active recombinant p38α in the presence of radioactive ATP (upper panel). Coomassie staining is shown in the lower panel. **f** Myc-ULK1 proteins either WT or the S555A mutant were overexpressed in HEK293 cells, immunoprecipitated and incubated with active recombinant p38α in the presence of cold ATP, and then were analyzed by immunoblotting with the indicated antibodies. **g** Colocalization of GFP-ULK1 (green) and Myc-p38α (red) at 8 h and 48 h after MKK6 induction. Bar = 10 μm. **h** Quantification of the colocalizing proteins in **g** at the indicated times. Differences between control and MKK6-expressing cells, or between the two groups indicated were analyzed using the unpaired Student's *t* test, (****) *p* < 0.00001, (**) *p* < 0.001, (*) *p* < 0.01

We first examined ULK1 implication in p38α-induced autophagy. We used siRNA to downregulate ULK1 (Fig. 3a), and found that reduced ULK1 expression decreased the numbers of GFP-LC3^+^ puncta in MKK6-expressing cells (Fig. 3b), suggesting that ULK1 is required for the p38α-induced autophagosome formation. Immunoblot analysis showed that Ser-555 phosphorylation, which is required for ULK1 activation^32^, was enhanced at 24 h after MKK6 induction but was impaired by either p38α inhibition (Fig. 3c) or p38α deletion (Fig. 3d). Next, we performed kinase assays using purified active p38α and ULK1 as a substrate. As ULK1 has a high auto-phosphorylation activity^33^, we used a T180A ULK1 mutant to reduce auto-phosphorylation. Our results indicated that p38α can phosphorylate ULK1 (Fig. 3e), and the use of phospho-specific antibodies identified Ser-555 as a site phosphorylated by p38α (Fig. 3f).

To support the link between p38α activation and ULK1 phosphorylation on Ser-555, cells were treated with ULK1 siRNA followed by expression of either WT ULK1 or the mutant S555A. We found that the endogenous levels of LC3^+^ puncta induced by MKK6 were significantly lower in cells expressing ULK1-S555A compared with cells expressing WT ULK1 (Supplementary Fig. 4). These results are consistent with the idea that Ser-555 phosphorylation of ULK1 contributes to autophagy induction by p38α.

Our results showed that the p38α pathway activity reached a peak 4–8 h after MKK6 induction (Fig. 1a and Supplementary Fig. 3A), whereas the enhanced autophagic flux was not detected until 24–48 h (Fig. 2b, c and Supplementary Fig. 3D), which was consistent with the enhanced ULK1 phosphorylation at 24–48 h (Fig. 3c, d). To address this difference between the timing of p38α activation and ULK1 phosphorylation, we analyzed the colocalization of cMyc-p38α with GFP-ULK1. We found that the colocalization of both proteins significantly increased 48 h after MKK6 induction compared to control cells or with cells expressing MKK6 for 8 h (Fig. 3g, h). Collectively, our results indicate that p38α can directly phosphorylate ULK1, but there is a delay in MKK6-expressing cells between p38α activation and ULK1 phosphorylation and autophagy induction.

### Autophagy induction by p38α requires mitochondrial ROS production

**Fig. 4:**
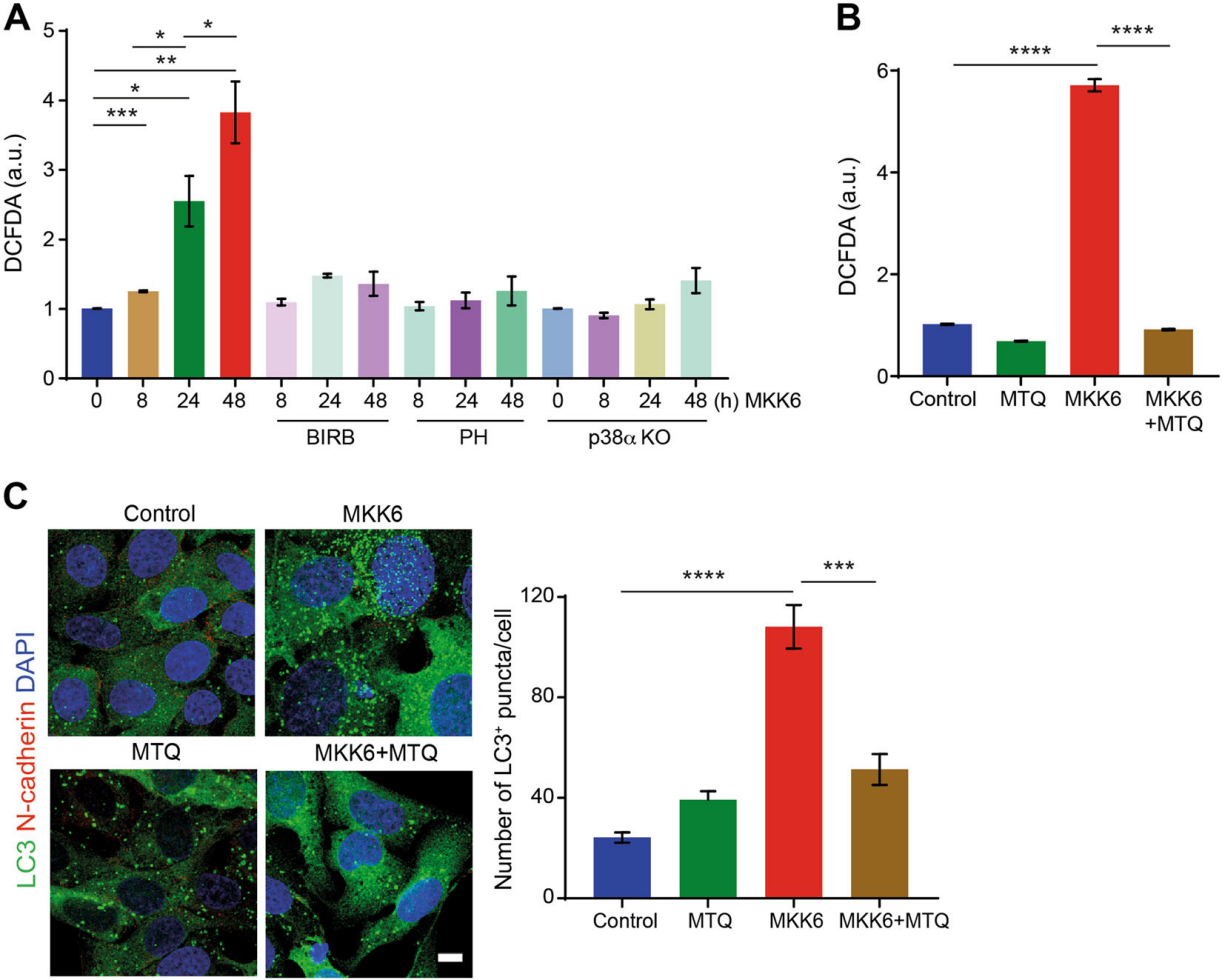
p38α-induced autophagy requires mitochondrial ROS production. U2OS cells expressing a Tet-regulated construct were either mock treated (control) or treated with tetracycline for the indicated times to induce the expression of constitutively active MKK6. **a** Total ROS levels were analyzed by FACS using the 2′,7′-Dichlorofluorescin diacetate (DCFDA) probe in MKK6-expressing cells treated with the p38α inhibitors PH797804 (PH) or BIRB796 (BIRB), or in p38α KO cells. Values are presented as fold change of mean fluorescence intensity of DCFDA versus the control cells (time 0). **b** Total ROS levels were analyzed by FACS using the DCFDA probe in cells expressing MKK6 for 48 h in the presence or absence of MitoQ (500 nm). **c** Cells treated as in **b** were analyzed for LC3^+^ puncta (autophagosomes) by immunofluorescence. Bar = 10 μm. The histogram shows the quantification of puncta. Differences between control and MKK6-expressing cells, or between the two groups indicated were analyzed using the unpaired Student's *t* test, (****) *p* < 0.00001, (***) *p* < 0.0001, (**) *p* < 0.001, (*) *p* < 0.01

Sustained p38α activation in U2OS cells induces metabolic changes, which lead to the enhanced production of mitochondrial reactive oxygen species (ROS)^26^. As mitochondrial ROS has been linked to autophagy^34^, we investigated its possible implication in p38α-induced autophagy. We confirmed that ROS levels started to increase 8 h after MKK6 induction, with further increases observed later, and this was impaired by p38α inhibitors or p38α downregulation (Fig. 4a). Treatment with the mitochondrial-specific antioxidant MitoQ^35^ rescued the enhanced ROS levels induced by MKK6 expression (Fig. 4b). Immunofluorescence analysis showed that the number of LC3^+^ vesicles in MKK6-expressing cells was reduced by MitoQ treatment (Fig. 4c), indicating that mitochondrial ROS production contributes to p38α-induced autophagy.

As mitochondrial ROS induced by MKK6 expression is generated through the p38α/MK2 axis^26^, we explored the implication of MK2 in p38α-induced autophagy. Immunoblot analysis confirmed that two different MK2 inhibitors partially impaired the MKK6-induced LC3 lipidation as well as ULK1 phosphorylation on Ser-555 (Supplementary Fig. 5A). Furthermore, the number of LC3^+^ puncta induced by MKK6 expression was reduced by MK2 inhibition to about control levels in both U2OS and Saos-2 cells (Supplementary Fig. 5B and C). These results support that p38α-induced autophagy requires mitochondrial ROS production mediated by MK2.

### Cells with sustained p38α activation can enter senescence or apoptosis

**Fig. 5:**
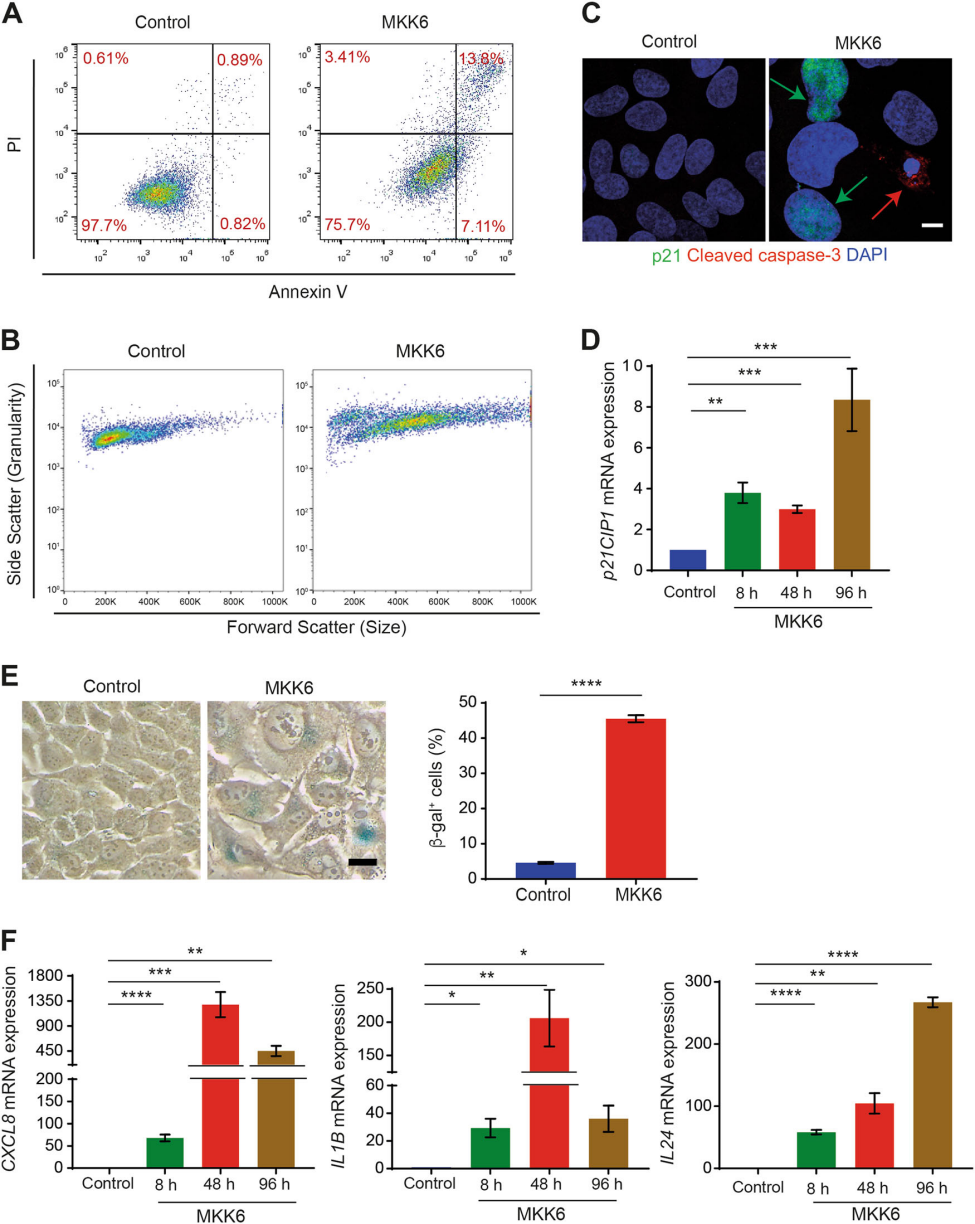
Sustained p38α activity can lead to senescence or apoptosis. U2OS cells expressing a Tet-regulated construct were either mock treated (control) or treated with tetracycline for the indicated times to induce the expression of constitutively active MKK6. **a** Cells expressing MKK6 for 48 h were analyzed by FACS using Annexin V/PI staining. **b** FACS analysis of cell size (forward scatter–horizontal) and granularity (side scatter–vertical). **c** Representative immunofluorescence images to illustrate the detection of p21^+^-senescent cells (green arrows) and cleaved caspase-3^+^ apoptotic cells (red arrow) in cells expressing MKK6 for 48 h. No co-expression of p21 and cleaved caspase-3 was observed in > 100 cells analyzed. Bar = 10 μm. **d** The expression levels of *CDKN1A* mRNA-encoding p21 gene were analyzed in cells treated as indicated. Results are presented as fold change versus the control. **e** Staining of senescent cells using β-gal after 48 h of MKK6 induction. Bar = 125 μm. The histogram shows the quantification of the senescent cells. **f** Expression levels of *CXCL8* (IL8*), IL1B* (IL1β) and *IL24* (IL24) mRNAs were analyzed in cells treated as indicated. Results are presented as fold change versus the control. Differences between control and MKK6-expressing cells were analyzed using the unpaired Student's *t* test, (****) *p* < 0.00001, (***) *p* < 0.0001, (**) *p* < 0.001, (*) *p* < 0.01

In agreement with a recent report^26^, activation of the p38α pathway for 48 h induces 25–40% of the U2OS cells to undergo cell death (Fig. 5a), raising the question of what was the status of the other MKK6-expressing cells. We found that MKK6 induction increased the cell size, as determined by microscopy examination (Supplementary Fig. 6A) and by Forward Scatter flow cytometry analysis (Fig. 5b). As increased cell size is a characteristic of senescent cells, we hypothesized that sustained p38α activation might lead to some cells entering senescence. Immunofluorescence analysis 48 h after MKK6 induction showed a clear distinction between caspase-3^+^ cells and p21^+^ cells, as markers of apoptosis and senescence, respectively (Fig. 5c). Reverse transcription polymerase chain reaction (RT-PCR) analysis confirmed the MKK6-induced upregulation of *CDKN1A* mRNA encoding p21 (Fig. 5d). Senescence-associated β-galactosidase (β-gal) staining showed that 35–40% of cells expressing MKK6 for 48 h were senescent (Fig. 5e). Senescent cells express higher levels of cytokines and chemokines^3,36^, and we observed by qRT-PCR enhanced expression of the mRNAs for *CXCL8* (IL8), *IL1B* (IL1β), and *IL24* (IL24) starting 8 h after MKK6 induction (Fig. 5f). These findings show that sustained p38α activity can lead to senescence or apoptosis.

### p38α-induced autophagy determines cancer cell fate

**Fig. 6:**
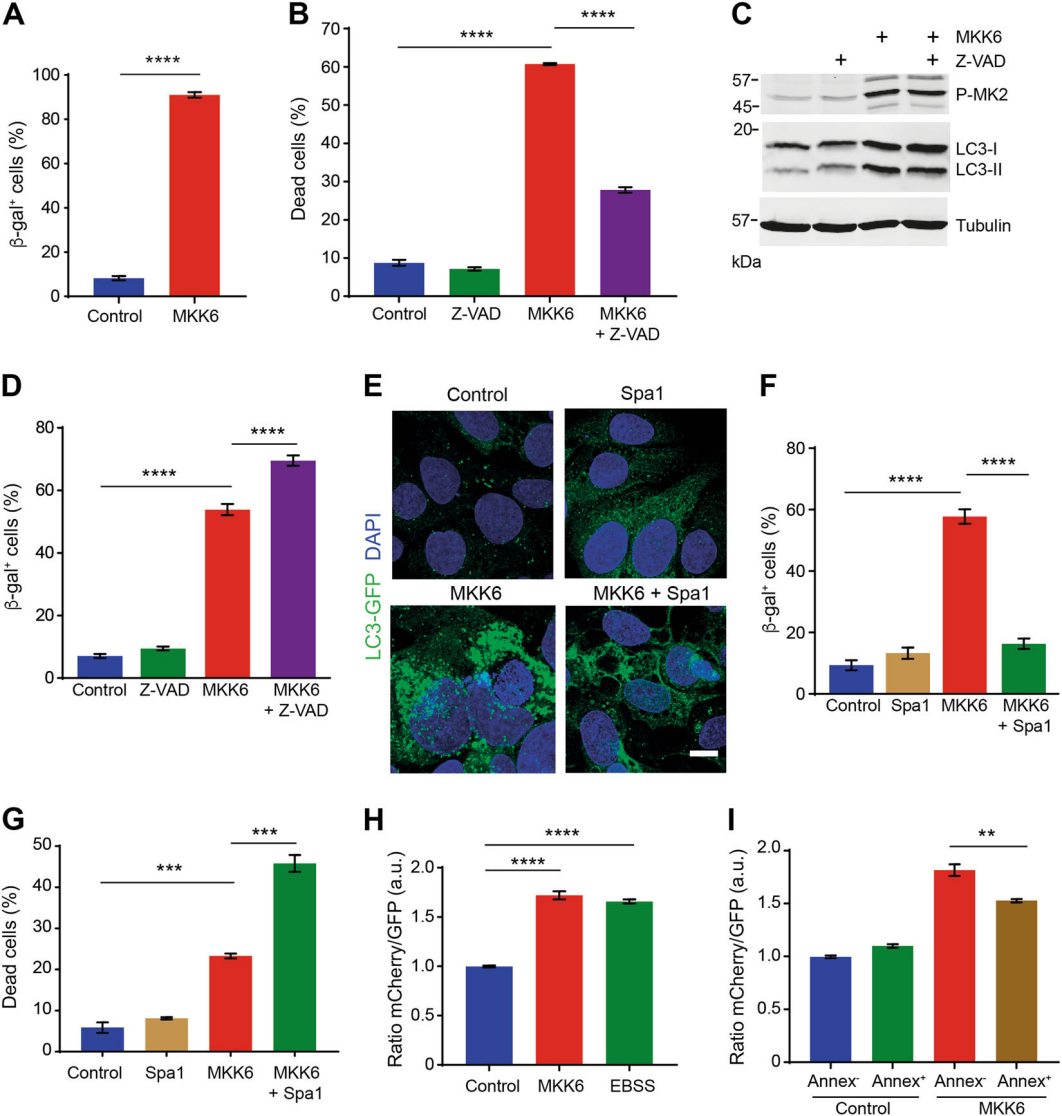
p38α-mediated autophagy determines cancer cell fate. U2OS cells expressing a Tet-regulated construct were either mock treated (control) or treated with tetracycline for the indicated times to induce the expression of constitutively active MKK6. **a** Quantification of senescent cells stained with β-gal at 96 h after MKK6 induction. **b** Cells expressing MKK6 for 96 h were assayed by FACS using Annexin V/PI staining. Cell were treated with the pan-caspase inhibitor Z-VAD (50 μm) as indicated every 24 h. Cell death was determined by FACS using Annexin V/PI staining. **c** Total lysates of cells treated as in **b** were analyzed by immunoblotting using the indicated antibodies. **d** Quantification of senescent cells stained for β-gal upon expression of MKK6 for 48 h in the presence or absence of Z-VAD. **e** Representative immunofluorescence images to illustrate the detection of GFP-LC3^+^ autophagosomes in cells expressing MKK6 for 48 h in the presence or absence of the autophagy inhibitor Spautin-1 (Spa1, 10 μm). **f** Quantification of senescent cells stained with β-gal in cells treated as in **e**. **g** Cells were treated as in **e** and cell death was determined by FACS using Annexin V/PI staining. **h** Ratiometric quantification of autophagic flux by FACS using the mCherry-GFP-LC3 reporter in cells expressing MKK6 for 48 h. As a positive control, cells were exposed to autophagy-inducing EBSS media for 8 h. **i** Ratiometric quantification of the autophagic flux was performed as in **h** in the Annexin V^+^ (Annex^+^) and Annexin V^−^ (Annex^−^) cell populations. Differences between control and MKK6-expressing cells, or between the two groups indicated were analyzed using the unpaired Student's *t* test, (****) *p* < 0.00001, (***) *p* < 0.0001, (**) *p* < 0.001

As U2OS cells with sustained p38α activity can enter senescence or apoptosis, we speculated that the disappearance of the apoptotic cells should eventually increase the percentage of senescent cells in the population. Consistent with this idea, 96 h after MKK6 induction the majority of the attached cells on the plate were β-gal^+^ (Fig. 6a). Treatment with the pan-caspase inhibitor Z-VAD reduced the number of dead cells observed 96 h after MKK6 induction (Fig. 6b), but did not restore cell morphology (Supplementary Fig. 6B) nor reduced the LC3-II levels (Fig. 6c), suggesting that apoptosis inhibition does not affect autophagy (Fig. 6c). When MKK6 was expressed for only 48 h, to avoid that the population was saturated with senescent cells, we found that Z-VAD treatment increased the number of senescent cells (Fig. 6d).

Next, we evaluated the role of the p53 protein in p38α-induced autophagy and cell fate determination. We used siRNA to downregulate p53 in U2OS cells and found that p53 silencing did not affect the LC3 lipidation levels observed 48 h after MKK6 induction (Supplementary Fig. 7A). Moreover, p53 silencing increased the number of dead cell induced by MKK6 expression (Supplementary Fig. 7B), without affecting the number of senescent cells (Supplementary Fig. 7C), indicating that, in our cell system, neither autophagy nor senescence are regulated by p53.

To test whether autophagy and senescence induction were linked, we used the autophagy inhibitor Spautin-1^37^. We confirmed that Spautin-1 decreased the number of GFP-LC3^+^ autophagosomes induced by MKK6 expression (Fig. 6e), as well as the number of senescent cells (Fig. 6f). These results connected p38α-induced autophagy with senescence entry in cancer cells. Moreover, autophagy inhibition significantly enhanced the levels of cell death induced by sustained p38α activation (Fig. 6g). Therefore, p38α-induced autophagy appears to function as a pro-survival response to stress that leads to senescence instead of apoptosis.

Our observations suggested that autophagy induction might determine whether cells die or enter senescence. To measure the autophagic flux in cells undergoing different fates, we quantified the signal from the mCherry-GFP-LC3 reporter by FACS. We found that MKK6 induction for 48 h increased the ratio of mCherry/GFP to a similar extent as the treatment with EBSS, which serve as a positive control (Fig. 6h). Then, we isolated by FACS Annexin^+^ and Annexin^−^ MKK6-expressing cells, and found that Annexin^+^ cells showed a lower level of autophagic flux than Annexin^−^ cells (Fig. 6i). These findings support the idea that stress-induced autophagy favors senescence over apoptosis. Taken together, our results indicate that the level of p38α-induced autophagy may determine whether cells undergo apoptosis or senescence in response to stress.

### p38α-induced autophagy regulates the response to chemotherapeutic drugs

**Fig. 7:**
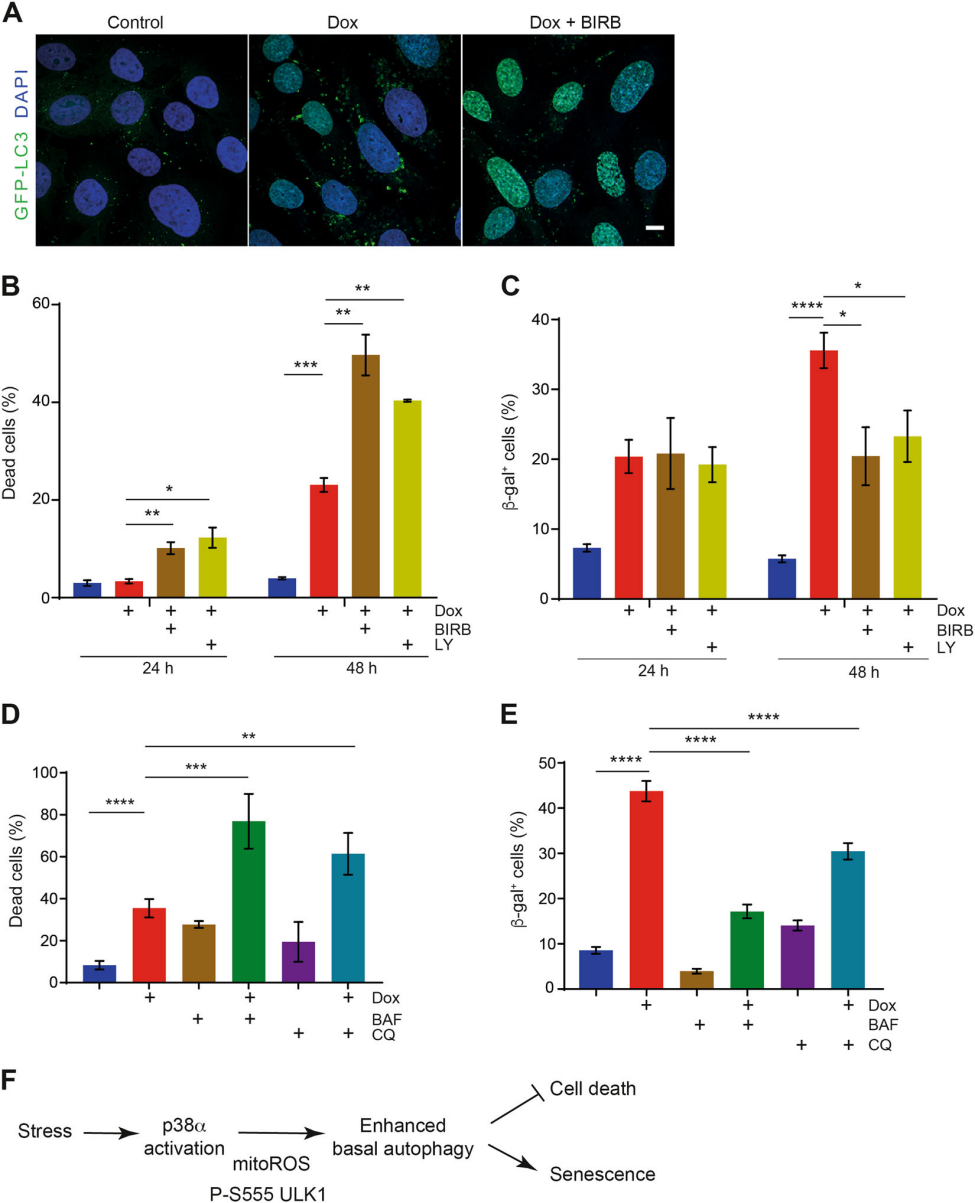
p38α determines cancer cell fate upon doxorubicin treatment. **a** Representative immunofluorescence images to illustrate the GFP-LC3^+^ puncta (autophagosomes) detected in U2OS cells treated with doxorubicin (Dox, 250 nm) for 24 h in the presence or absence of the p38α inhibitor BIRB796 (BIRB). Bar = 10 μm. **b** U2OS cells were treated with Dox for 24 h and 48 h in the presence or absence of the p38α inhibitors BIRB and LY2228820 (LY) and cell death was determined by FACS using Annexin V/PI staining. **c** U2OS cells treated as in **b** were analyzed for senescence by staining with β-gal. **d** U2OS cells were treated with Dox for 48 h in the presence or absence of the autophagy inhibitors bafilomycin A1 (BAF, 50 nm) and chloroquine (CQ, 20 μm), which were added 24 h after the Dox treatment started, and cell death was determined by FACS using Annexin V/PI staining. **e** U2OS cells treated as in **d** were analyzed for senescence by staining with β-gal. **f** Scheme summarizing the mechanism of autophagy-induced cell fate determination by p38α. Stress conditions lead to the activation of the p38α pathway, which increases mitochondrial ROS levels. Enhanced mitochondrial ROS above a certain threshold, trigger the phosphorylation of ULK1 on Ser-555 by p38α, which increases the autophagic flux leading to the cells entering into senescence instead of apoptosis. Differences between control and treated cells were analyzed using the unpaired Student's *t* test, (****) *p* < 0.00001, (***) *p* < 0.0001, (**) *p* < 0.001, (*) *p* < 0.01

To validate our findings in pathophysiological conditions, we focused on doxorubicin, a chemotherapeutic drug that at low doses can induce senescence and cell death in a time-dependent manner^5,38,39^. We found that U2OS cells treated with doxorubicin for 24 h showed increased autophagosome production, which was reduced by p38α inhibition (Fig. 7a). The ability of doxorubicin to induce cancer cell death was potentiated by p38α inhibition in both U2OS cells (Fig. 7b) and A549 cells (Supplementary Fig. 8A). However, doxorubicin-induced senescence was either unaffected (24 h) or decreased (48 h) by p38α inhibition (Fig. 7c). Similar results were observed in U2OS cells treated with camptothecin or palbociclib, whose ability to induce cancer cell death was also potentiated by p38α inhibition (Supplementary Fig. 8B and C).

To test the hypothesis that p38α-induced autophagy determines cancer cell fate upon chemotherapeutic drug treatment, we combined doxorubicin with low concentrations of the autophagy inhibitors bafilomycin A1 or chloroquine, which were added only for the last 24 h of doxorubicin treatment to reduce the cell death observed upon long term treatment with these compounds^40^. We found that chloroquine or bafilomycin A1 induced some cell death, but their combination with doxorubicin substantially increased the number of dead cells (Fig. 7d). The pro-survival role of autophagy in response to doxorubicin was confirmed using cells treated with ATG5 siRNA to downregulate ATG5, which showed increased susceptibility to doxorubicin-induced cell death (Supplementary Fig. 8D). In contrast, the combination of autophagy inhibition with doxorubicin reduced the number of senescent cells in comparison with doxorubicin alone (Fig. 7e). Overall, our data support that p38α-induced autophagy favors the entry into senescence of doxorubicin-treated cells while inhibition of either p38α or autophagy increases cell death, implicating the control of autophagy by p38α in the modulation of cancer cell fate in response to doxorubicin.

## Discussion

Senescence and apoptosis are two mutually exclusive responses to various stresses, though it is unclear how cells make the choice. Autophagy is often associated with stress, and has been linked to both apoptosis and senescence^12,41^. Here, we show that specific p38α activation induces autophagosome formation and increases the autophagic flux, leading the cells to enter senescence instead of apoptosis.

There is evidence that p38α can activate chaperone-mediated autophagy by phosphorylating the lysosomal receptor LAMP2A in response to endoplasmic reticulum stress^42^. However, p38α normally inhibits both basal and starvation-induced macroautophagy, either through binding to p38IP, an essential mAtg9 interactor during autophagosome formation^23^, or by phosphorylating Atg5^22^. Recently, p38α has been reported to inhibit autophagy by phosphorylating ULK1 on Ser-757^21^, a site that negatively regulates ULK1 and is usually phosphorylated by mTOR kinase^43^. However, ULK1 phosphorylation on Ser-757 by p38α seems to be uniquely associated with microglial cells^21^. In contrast, our results illustrate a mechanism by which sustained p38α signaling can induce macroautophagy, which involves ULK1 phosphorylation on the activatory site Ser-555.

The observed delay between p38α activation and the phosphorylation of ULK1 in cells suggests the need for additional signals to initiate autophagy. Sustained p38α activation can increase mitochondrial ROS production^26^, which we found contributes to p38α-induced autophagy and somewhat facilitates p38α interaction with ULK1 and its phosphorylation on Ser-555. Consistent with previous reports connecting oxidative stress and autophagy initiation^44,45^, we speculate that mitochondrial ROS might serve as a threshold sensor for cell damage, leading to autophagy induction once it reaches some critical level. Mitochondria are considered the main source of ROS in the regulation of autophagy, whereas NADPH oxidase contributes to the activation of selective xenophagy^45^. Mechanistically, mitochondrial ROS can stimulate autophagy through the inhibitory oxidation of the cysteine protease ATG4, which in turn inhibits de-lipidation of LC3^45^, and can activate lysosomal TRPML1 channels, inducing lysosomal Ca^2+^ release with lysosome biogenesis^46^. Increased ROS levels can also activate various autophagy-regulating transcription factors such as HIF-1, p53, and FOXO3, or may induce autophagy via AMPK activation and mTOR inhibition^47^. Our data implicate mitochondrial ROS induced by p38α signaling as an essential regulator of stress-induced autophagy. We also provide evidence that p53 does not play a major role in this process.

It seems likely that cells interpret the hyperactivation of p38α as a signal of constant stress. In fact, sustained p38α activation has been reported to induce mitochondrial changes that may eventually result in apoptotic cell death^26^. Interestingly, we observed that although some cells with hyperactive p38α indeed undergo cell death others enter into senescence. The links between senescence, apoptosis, and autophagy are complex and in some cases cell type specific^2,48,49^. We found that senescent cells have a higher autophagic flux, suggesting a potential adaptive strategy of cancer cells to avoid cell death in response to limited stress-induced damage. A key question is how cells that presumably express similar amounts of MKK6, and hence activate the p38α pathway to similar extents, achieve different levels of autophagic flux. We speculate that p38α activity levels might not be the same in all the cells, either owing to variability in MKK6 expression levels, or to other factors that directly or indirectly affect activation of the p38α pathway. Another possibility would be that variability in cell–cell interactions among different cells in the population combined with the hyperactivation of p38α impinges on cell fate. For example, gap junction-mediated cell–cell communication may induce senescence^50^.

Our observation that autophagy suppression reduces entry of cells with high p38α activity levels into senescence while increasing the number of dead cells, is consistent with the view of autophagy as an anti-apoptotic mechanism in cancer cells under stress^51^, and the evidence that autophagy can promote senescence^41^. Interestingly, we found that suppression of apoptosis in cells with p38α hyperactivation increases the number of β-gal^+^ cells, confirming that high autophagy levels eventually promote senescence if cells do not die.

To address the importance of our findings in pathophysiological conditions, we used doxorubicin, a drug widely employed for cancer treatment that induces apoptosis but at low doses can also trigger senescence^4,6^. We show that doxorubicin increases the number of autophagosomes in a p38α-dependent manner, and inhibition of either p38α or autophagy enhances doxorubicin-induced cell death, with a concomitant decrease in the number of senescent cells. These observations extend previous reports, proposing that autophagy inhibition may redirect cancer cells treated with drugs like temozolomide or with radiation from senescence to apoptosis^52,53^. Our results support that p38α activation engages autophagy as a pro-survival mechanism, determining the fate of cancer cells treated with doxorubicin or other drugs.

In conclusion, we describe a new mechanism based on mitochondrial ROS accumulation and ULK1 phosphorylation on Ser-555, by which p38α can induce autophagy and influence cancer cell fate decisions in response to stress (Fig. 7f). We provide evidence that p38α-induced autophagy can modulate whether cancer cells enter senescence or apoptosis in response to drug treatment. Overall, elucidating the cellular contexts in which these mechanisms apply may contribute to improve cancer treatments.

## Materials and methods

### Materials

The following commercially available antibodies were used: p38α (Cell Signaling, 9218), phospho-MAPKAPK2/MK2 (Thr334) (Cell Signaling, 3007 S), MK2 (Cell Signaling, 3042), phospho-Hsp27 (S82) (Cell Signaling, 2401), Hsp27 (Santa Cruz, 1049), LC3 (MBL, PM036), p62 (BD, 610832), p53 (BD, 554294), N-cadherin (BD, 610920), LAMP1 (Santa Cruz, sc-18821), p21 (Santa Cruz, sc-397), CD63 (Thermo Fisher, MA1–19281), cleaved caspase-3 (Cell Signaling, 9661 S), ULK1 (Cell Signaling, 8054), phospho-ULK1 (Ser-555) (Cell Signaling, 5869 S), GFP (Abcam, ab6673), Myc tag (Abcam, ab9106), and Tubulin (Sigma, T9026). The MKK6 rabbit antiserum^54^ and the p38γ antibody^55^ have been previously described.

### Cell culture

U2OS, A549, Saos-2, and HEK293 cells (purchased from ATCC) were cultured either in Dulbecco’s modified Eagle medium (Sigma, D5796) or Roswell Park Memorial Institute Medium (RPMI)-1640 supplemented with 10% fetal bovine serum (Thermo Scientific, E6541L), 2 mm l-Glutamine (LabClinics, M11–004) and 100 μg/ml penicillin–streptomycin (LabClinics, P11–010). For the inhibition of p38α, we used 1 μm PH797804 (Selleckchem, S2726) or 500 nm BIRB0796 (Axon MedChem, 1358), and 200 nm LY2228820 (Selleckchem, S1494). MK2 was inhibited using 10 μm MK2 Inhibitor III (Calbiochem, 475864–5MG) or 10 μm PF 3644022 (Sigma, PZ0188). The following additional treatments were used: 0.5 μm MitoQ (kindly provided by M. Murphy, Cambridge, UK), 250 nm Doxorubicin hydrochloride (Sigma, D1515–10mg), 50 μm Z-VAD (OMe)-FMK (SM Biochemicals LLC SMFMK001), 200 or 50 nm bafilomycin A1 (Santa Cruz, sc-201550), 20 μm chloroquine (Sigma, C6628), 10 μm Spautin-1 (Axon MedChem, 2512), 15 μm palbociclib (Selleckchem, S1116), 500 nm camptothecin (Sigma, C9911). The compounds were dissolved in dimethyl sulfoxide (DMSO) or water and the total concentration of DMSO in the culture medium never exceeded 1%.

### Measurement of the autophagic flux

The autophagic flux was measured by several independent methods as described^30,56^. We determined the autophagic flux by immunoblotting based on the quantification of the LC3-II band. After antibody incubation, membranes were scanned and signal intensity was quantified using the Odyssey Infrared Imaging System, software version 2.0 (LI-COR Biosciences) according to the manufacturer’s protocol. The quantification of the LC3-II bands was done with median background subtraction and normalized to tubulin. Basal values were normalized to 1. Data are expressed as means ± standard errors of the means from three experiments. The difference in the amount of LC3-II between cells treated with or without bafilomycin A1 for 4 h represented the level of autophagic flux. We also used two different protocols based on LC3 puncta to measure autophagy flux. In the first one, a GFP-LC3 expressing cell line was generated by transfection of U2OS cells with the GFP-LC3 construct provided by C. Mauvezin (University of Minnesota, USA), followed by sorting using the LSR-II (BD Biosciences) cytometer in order to get a homogenous cell population, which was grown in media with 500 μg/ml G418 (Sigma, G8168). These cells were treated for 4 h with bafilomycin A1 or DMSO (control) and GFP-LC3 fluorescence levels were analyzed by FACS. Results were expressed as the change in GFP-LC3 MFI in bafilomycin-treated compared with untreated cells^14^. In the second approach, cells were transduced with a pBABE-puro retrovirus expressing mCherry-GFP-LC3B tandem fluorescent probe (Addgene #22418) and then were selected with 2 μg/ml puromycin. The autophagic flux was quantified either by confocal microscopy or by FACS. To measure the autophagic flux by confocal microscopy, cells were analyzed for the presence of GFP^+^ (green) or mCherry^+^ (red) puncta and the number of green puncta were subtracted from the number of red ones. Puncta detection was performed using Fiji macros. In brief, custom-made macros detected the nuclei in the 4′,6-diamidino-2-phenylindole (DAPI) channel, mCherry and GFP signals in red and green channels, respectively. Then, the number of dots and nuclei were quantified. At least, 25 cells in each experiment were quantified. As a control, we used two different conditions to induce or inhibit the autophagic flux. To induce autophagy and increased flux, cells were treated 8 h with EBSS (Thermo Fisher, 24010043), which contains no nutrients or growth factors and thus induces the autophagic flux. This resulted in the accumulation of red puncta, as when autophagosomes containing mCherry-GFP-LC3 fuse with lysosomes, the acidic pH of lysosomes quenches the GFP signal, releasing only the stable mCherry signal that is red. To induce inhibition of autophagic flux, cells were treated for 4 h with bafilomycin A1, a specific inhibitor of vacuolar type H^+^-ATPase, which increased the pH of lysosomes. In this case, once autophagosomes containing mCherry-GFP-LC3 fuse with lysosomes, the GFP part of the reporter that is sensitive to acidic pH cannot be quenched, owing to the increased pH of lysosomes upon bafilomycin A1 treatment, releasing in addition to the red signal (mCherry), the green one (GFP), which usually is stronger resulting in the yellow–green puncta. To measure the autophagic flux by FACS, the median mCherry/GFP ratio was analyzed as described earlier^30^ using LSR-II (BD Biosciences) cytometer. As a positive control, cells were treated for 8 h with EBSS.

### Generation of Saos-2 cells expressing constitutively active MKK6

Saos-2 cells were transfected with the constructs pcDNA4 TO (Invitrogen) and pcDNA6-MKK6DD using NanoJuice (Merc Millipore). MKK6DD is a constitutively active form of the specific p38 MAPK activator MKK6 with the activation loop residues Ser207 and Thr221 mutated to Asp. After transfection, cells were trypsinized, plated at dilutions of 1:4, 1:3 and 1:2, and grown in media containing 4μg/ml Blasticidin S HCl (Invitrogen, A11139–03) and 35 μg/ml of Zeozin (InvivoGen, ant-zn-1). Media was changed every 3 days and when colonies were large enough, they were picked and replated in a 24-well plate. Selected clones were amplified and tested for p38 MAPK activation. To induce MKK6DD expression, cells were plated at 2.5 × 10^5^ for a six-well plate, 5 × 10^5^ for a 60-mm plate or 1.2 × 10^6^ for a 10-cm plate and when they reached 85% confluence, fresh media containing 1 μg/ml tetracycline (Sigma 87128–25 G) or the corresponding amount of ethanol (1:1000 dilution) was added. Tetracycline stocks were prepared in ethanol at 1 mg/ml and stored at −20 °C.

### Immunoprecipitation

Cells were lysed in buffer containing 50 mm Tris-HCl pH 7.5, 150 mm NaCl, 1% NP-40 (Igepal), 5 mm EGTA, 5 mm EDTA, and protease and phosphatase inhibitor cocktails (PhosSTOP^TM^ cat. 4906845001 and cOmplete^TM^, cat. 11697498001 Roche). To immunoprecipitate Myc-tagged ULK1, we used anti-c-Myc agarose beads (Sigma, A7470–1ML) following the manufacturer’s indications.

### Senescence-associated-β-gal staining

The Senescence β Galactosidase staining kit (Cell Signaling 9860) was used to determine cellular senescence following the manufacturer’s indications. The blue-stained cells were counted in 10 fields under the inverted microscope Nikon Eclipse TE-200 with × 200 magnification and positive cells were expressed as a percentage of the total number of cells counted.

### Transfection with siRNA

Cells were grown up to 60% confluence in 60 mm dishes and were transfected using Dharmafect transfection kit according to the manufacturer’s instruction with 4 μl of 25 μm siRNAs for ULK1 (ID HSS140824, Thermo Fisher, cat. 1299001), p53 (ID 106141, Thermo Fisher, cat. AM51331) or ATG5 (ID HSS114103, Thermo Fisher, cat. 1299001) to the final concentration of 100 nm. Cells were plated 48 h later and left to recover for 16 h before proceeding with MKK6 induction or drug treatments. For ectopic ULK1 expression, 48 h after transfection with ULK1 siRNA, cells were transfected with ULK1-expresing plasmids and 24 h later MKK6 was induced.

### Annexin V/PI staining

Cell death was assayed using the luorescein isothiocyanate (FITC) Annexin V kit (BD Pharmingen, 556547). The day before the experiment, 2 × 10^5^ cells/well were plated on a six-well dish. After appropriate treatments, media was collected into 2 ml Eppendorf tube. The cell monolayer was washed once with phosphate-buffered saline (PBS) and 200 μl of trypsin was added. Cells were collected using the previously removed media. The whole population of dead and live cells was centrifuged for 5 min at 1000 rpm. Cells were washed once with 1 × Binding buffer and re-suspended in 500 μl of binding buffer at a concentration 1 × 10^6^ cells/ml. In total, 100 μl of the solution (1 × 10^5^ cells) was transferred into a new Eppendorf tube and 4.5 μl of FITC Annexin V were added. Samples were incubated for 20 min at room temperature (RT) in the dark, after which 450 μl of binding buffer and 5 μl of PI solution was added. Samples were transferred to ice and analyzed immediately using Gallios Flow Cytometer (Beckman Coulter). Cell death was determined as the sum of cell populations that were Annexin V^+^/PI^−^, Annexin V^+^/PI^+^ and Annexin V^−^/PI^+^.

### ROS detection

To detect ROS production, the 2’,7’-dichlorofluorescin diacetate (DCFH-DA) (Sigma, D6883) was used. One day prior to the treatments, 2 × 10^5^ cells were plated per well in a six-well dish. To measure ROS, DCFH-DA (final concentration 10 μm) was added to the media for the last 30 min. After the incubation media was removed, cells were washed once with PBS, trypsinized and re-suspended in 500 μl of PBS containing 1.4 μg/ml aprotinin. Analysis was performed immediately by flow cytometry using Gallios Flow Cytometer (Beckman Coulter).

### mRNA expression analysis

Total RNA was extracted from cells using the RNA mini kit from Ambion according to the manufacturer’s instructions. To determine RNA concentrations, the absorbance at 260 nm was measured. Complementary DNA (cDNA) was obtained from 1 μg of the purified RNA using SuperScript IV Reverse Transcriptase (Invitrogen) in a final volume of 20 μl. For RT-PCR, 4 μl of cDNA (50 ng/reaction) were loaded in triplicates in a 96-well plate and 6 μl of the reaction mix were added. The plate was sealed and centrifuged for 1 min at 200 × *g* and run as follows: 50 °C for 2 min, 95 °C for 10 min, 40 cycles of denaturation at 95 °C for 15 s, annealing at 56 °C for 15 s, elongation at 72 °C for 60 s, and three final steps of 95 °C for 15 s, 60 °C for 2 min and 95 °C for 15 s. Glyceraldehyde-3-phosphate dehydrogenase was used as a reference and the ΔΔC(t) method was used to quantify gene expression. The primer sequences are presented in Supplementary Table 1.

### Immunoblotting

Total cell lysates (50 μg) were separated on 8, 12, or 14% sodium dodecyl sulfate–polyacrylamide gel electrophoresis (SDS–PAGE) Laemmli gels, depending on the molecular weight of the protein of interest. A wet-blotting system (Bio Rad) was used for protein transfer to nitrocellulose membrane, which were stained with 0.1% Ponceau (in 5% acetic acid) to evaluate transfer efficiency. Membranes were blocked for 1 h at RT in 5% non-fatty milk (in PBS). Primary antibodies were diluted in PBS with 5% BSA and 0.1% Tween 20. The antibodies were used at a concentration of 1:1000, except anti-tubulin that was used at 1:5000 as a loading control. Secondary Alexa Fluor-conjugated antibodies were diluted 1:5000 in 1% non-fatty milk. Membranes were analyzed using the Odyssey Infrared Imaging System (Li-Cor Biosciences).

### Generation of knockout cell lines

Forward and reverse oligonucleotides containing the CRISPR guide sequences (Supplementary Table 2) for the genes encoding p38α (*MAPK14*) and p38γ (*MAPK12*) were annealed and cloned into the pX330 plasmid^57^, which was subsequently transfected into U2OS cells using the NanoJuice™ Transfection Kit (Novagen). GFP^+^ cells were sorted 24 h after transfection (BD FACSAria Fusion Cell Sorter) and were grown as single clones. Screening for p38α KO and p38γ KO cells was carried out by immunoblotting. Two U2OS cell clones showing undetectable levels of p38α or p38γ proteins were selected for further analysis.

### Retroviral production and transduction

For retroviral production, 6 μg of pBabe-puro mCherry-GFP-LC3 (Addgene #22418) or pBabe-puro-EGFP (enhanced green fluorescent protein) as a control were mixed with 0.6 μg of pV-SVGR and 5.4 μg of pGAG-POL packaging vectors (provided by Roger Gomis, IRB Barcelona) in NaCl (150 mm) supplemented with PEI (5.8 μg/ml)^58^. HEK293T cells were plated on a 100 mm tissue culture dish, grown overnight, and transfected with the plasmid mix for further viral production at 37 °C for 48 h. The supernatant of HEK293T cells was collected and filtered through polyvinylidene difluoride filter (0.45 μm). Retroviral transduction was performed with 5 ml of media containing viral particles mixed with 5 ml of fresh media supplemented with 8 μg/ml polybrene (Sigma, 107689) in a 100 mm dish with the recipient cells for 24 h. Medium was then replaced and the cells were selected in the presence of 2 μg/ml puromycin (Sigma, P9620).

### Expression of Myc-ULK1 in HEK293T cells

To express the Myc-ULK1 (human) protein, HEK293T cells were transiently transfected with 5 μg of plasmid (Addgene #31961) supplemented with 50 μl of 2.5 m CaCl_2_, and MQ water up to 500 μl. After 5 min of incubation at RT, 500 μl of 2× HBS were added drop-wise, mixed, and incubate for 20 min at RT. The mixture was added drop-wise to the HEK293T cells and the next day the media was refreshed, leaving cells up to 48 h.

### Mutagenesis of Myc-hULK1

To generate the phosphorylation site point mutations T180A and S555A in Myc-hULK1, we used the Quick Mutagenesis Kit from Promega following manufacturer’s instructions and the following primers (mutated nucleotides are underlined): for T180A, Fw-CATGATGGCGGCCGCACTCTGCGGCTC and

Rv-GAGCCGCAGAGTGCGGCCGCCATCATG; for S555A Fw-CTGCCGCCTGCACGCCGCCCCCAACCTG and

Rv-CAGGTTGGGGGCGGCGTGCAGGCGGCAG.

The mutations were verified by DNA sequencing (Supplementary Table 3).

### Kinase assays

For in vitro kinase assays, Myc-hULK1 proteins were immunoprecipitated from 1 mg of HEK293 total cell lysates and were incubated for 45 min at 37 °C in a final volume of 20 μl of kinase buffer containing 50 mm Tris-HCl pH 7.5, 10 mm MgCl_2_, 1 mm DTT, 3 μm cold ATP, 1 mm NaF, 1 mm Na_3_VO_4_, 200 μμ phenylmethylsulfonyl fluoride, 10 μg/ ml each of aprotinin, leupeptin, and pepstatin, with 5 μCi of [γ-^32^P]ATP (Perkin Elmer) and 0.1 μg of bacterially expressed GST-p38α activated with MKK6^59^. The reactions were stopped by adding sample buffer and boiling 5 min, and were analyzed by SDS–PAGE and autoradiography. For cold kinase assays, immunoprecipitated Myc-hULK1 proteins were incubated with active GST-p38α (0.1 μg) as above but with 200 μμ cold ATP instead of [γ-^32^P]ATP. Proteins were resolved by SDS–PAGE, transferred onto a nitrocellulose membrane, and immunoblotted with phospho-Ser-555 ULK1 antibodies.

### Immunostaining and confocal microscopy

Cells were grown on coverslips, treated as indicated and then washed with PBS twice and fixed in 4% paraformaldehyde in PBS for 20 min, followed by washing with PBS once, permeabilization in 1% (v/v) Triton X-100 in PBS for 10 min and further washed with PBS containing 0.02% Tween 20 and 1% of BSA for 10 min. Then the coverslips were incubated with primary antibodies in blocking buffer (PBS with 3% BSA) in a humidified chamber for 1 h at 37 °C. Coverslips were washed with PBS containing 0.02% Tween 20 and 1% of BSA for 10 min and subsequently incubated with 1:300 Alexa-conjugated secondary antibodies (Invitrogen) for 45 min in humidified chamber for 1 h at 37 °C. Finally, coverslips were washed with PBS containing 0.02% Tween 20 for 5 min and once with PBS only and mounted using ProLong Gold Antifade Mountant with DAPI (Life Technologies, P36935). Confocal images were taken on the Leica TCS SPE microscope (Leica, Mannheim, Germany) using an HCX PL APO lambda blue × 63/1.3 oil immersion objective, 405-, 488-, 543-nm laser excitation at a pixel resolution of 71 nm. Z-stacks were acquired every 500 nm, to ensure a count of the puncta over the whole cell volume. The percentage of cells with GFP-LC3^+^ or endogenous LC3^+^ puncta structures was obtained by counting at least 25 cells in each working condition from three independent experiments. EGFP puncta detection was performed using Fiji macros. In brief, tailor-made macros segmented the nuclei (DAPI channel) and EGFP signal (green channel). Then, the number of dots and nuclei were automatically quantified. Endogenous LC3 puncta detection was performed using Fiji tailor made macros. As the cell membrane borders were hard to define with N-cadherin signal (red channel), our macros segmented the nuclei (DAPI channel), and LC3 signal (green channel). Then, after filtering and thresholding, the number of dots and nuclei were quantified. The number of puncta/cell is the result of dividing the total number of puncta by the total number of cells. This average value should not be very different from the one obtained in a cell by cell analysis. It is also relevant to mention that the LC3 signal was very specific, and was always found within the N-cadherin defined cell limits.

To investigate the colocalization of ULK1 with p38α, cells were grown on coverslips and co-transfected with GFP-ULK1 (provided by N. Mizushima, The University of Tokyo, Japan) and Myc-p38α^60^ plasmids. After the immunofluorescence performed as described above, colocalization was analyzed using a custom-made Fiji (ImageJ) macro. In brief, the macro built up two masks, one thresholding Myc-p38α signal in the red channel (R) and another thresholding the green signal (G) from GFP-ULK1. Then, colocalization areas were obtained with the Image Calculator using an “and” operation. This was done slice by slice along the Z-stack. Finally, the G/R ratio was obtained.

### Statistical analysis

All the statistics were performed using GraphPad Prism software using Student's *t* test unpaired two-tailed analysis: (****) *p* < 0.00001, (***) *p* < 0.0001 (**), *p* < 0.001, (*) *p* < 0.01.

## Supplementary information

Supplementary Information
