# Supplementary material for: Autophagy-induced senescence is regulated by p38α signaling
Source: Cell Death Dis. 2019 May 15;10(6):376. doi: 10.1038/s41419-019-1607-0 (PMC6520338; doi:10.1038/s41419-019-1607-0)
Supplement: Supplementary file 1 — Supplementary Information [file 41419_2019_1607_MOESM1_ESM.pdf]

Supplementary Information

Autophagy-induced senescence is regulated by p38α signaling

Konstantin Slobodnyuk, Nevenka Radic, Saška Ivanova, Anna Llado, Natalia Trempolec, Antonio Zorzano and Angel R. Nebreda

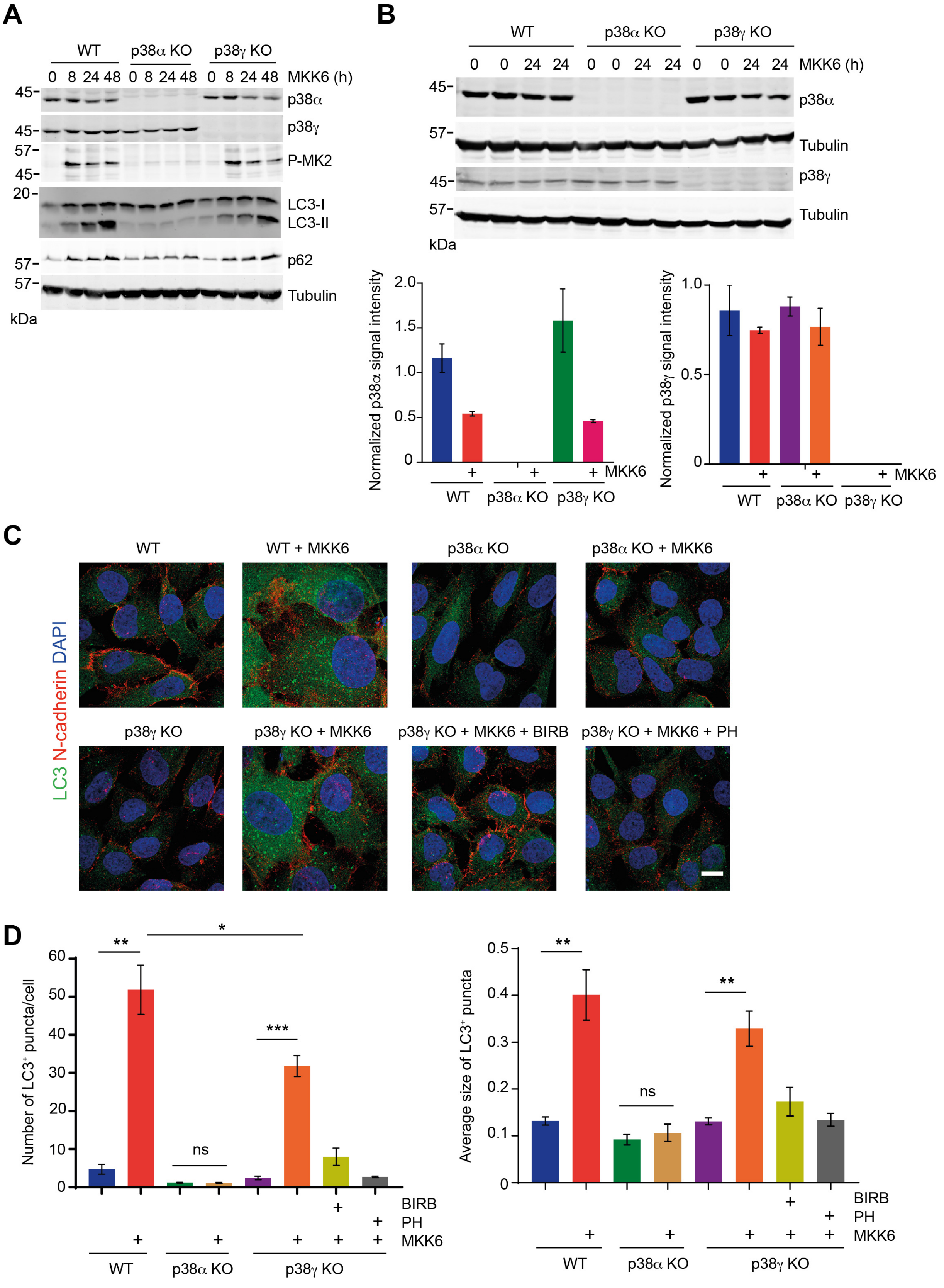

Supplementary Fig. 1. p38α plays a major role in the induction and maintenance of autophagy by MKK6. U2OS cells expressing a Tet-regulated construct were either mock treated (control) or treated with tetracycline for the indicated times to induce the expression of constitutively active MKK6. **(A)** Cells knockout for either p38α (p38α KO) or p38γ (p38γ KO) were generated from the U2OS cells expressing inducible MKK6 by using CRISPR/Cas9. Total lysates of the indicated KO or wild type (WT) cells were analyzed by immunoblotting at the indicated times after MKK6 induction. (**B**) Total lysates from cells WT, p38α KO or p38γ KO either expressing MKK6 or not, were analyzed by immunoblotting using the indicated antibodies and the bands were quantified using LICOR Odyssey software. (**C**) Detection of $LC3^+$ puncta (autophagosomes) by immunofluorescence in WT, p38α KO or p38γ KO cells expressing MKK6 for 24 h in the presence or absence of the p38α inhibitors PH797804 (PH) or BIRB796 (BIRB). Bar = 10 μm. (**D**) Quantification of $LC3^+$ puncta number and average size at 24 after MKK6 induction. Differences between control and MKK6 expressing cells or between the two groups indicated were analyzed using the unpaired Student t test, (***) $p < 0.0001$, (**) $p < 0.001$, (*) $p < 0.01$, ns, not significant.

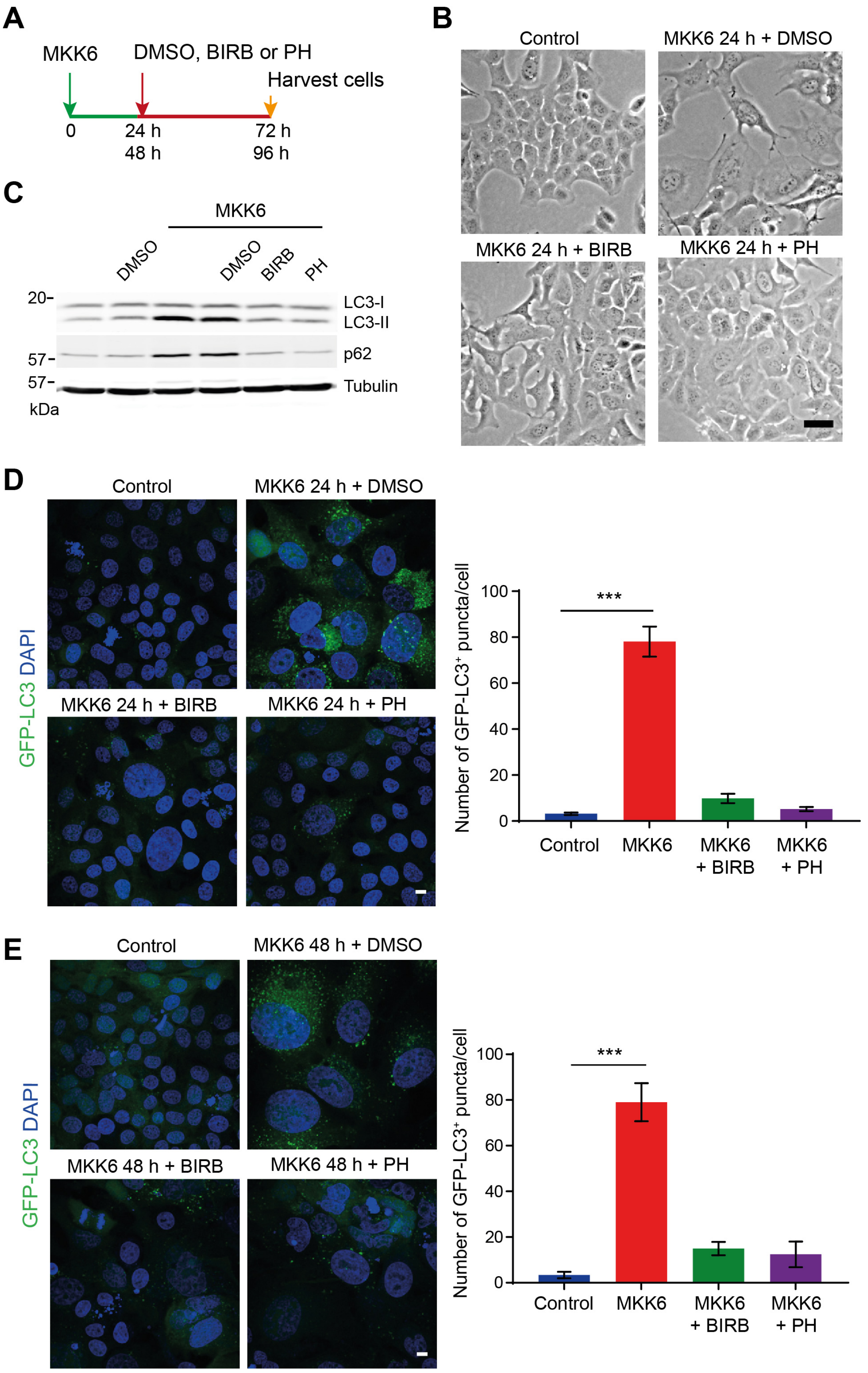

Supplementary Fig. 2. Sustained p38α activity is required for autophagy. U2OS cells expressing a Tet-regulated construct were either mock treated (control) or treated with tetracycline for the indicated times to induce the expression of constitutively active MKK6.

(**A**) Scheme indicating the experiments performed in cells expressing MKK6 for 24 h or 48 h and the subsequent change to media with DMSO or the p38α inhibitors PH797804 (PH) and BIRB796 (BIRB) for another 48 h. (**B**) Morphology of cells expressing MKK6 for 24 h followed by treatment with PH or BIRB for 48 h. Bar = 125 µm. (**C**) Total lysates of cells treated as in (**B**) were analyzed by immunoblotting using the indicated antibodies. (**D**) Detection of GFP-LC3$^+$ puncta in cells treated as in (**B**). Bar = 10 µm. The histogram shows the quantification of GFP-LC3$^+$ puncta. (**E**) Detection of GFP-LC3$^+$ puncta in cells expressing MKK6 for 48 h followed by treatment with PH or BIRB for another 48 h. Bar = 10 µm. The histogram shows the quantification of GFP-LC3$^+$ puncta. Differences between control and MKK6 expressing cells were analyzed using the unpaired Student t test, (***) $p < 0.0001$.

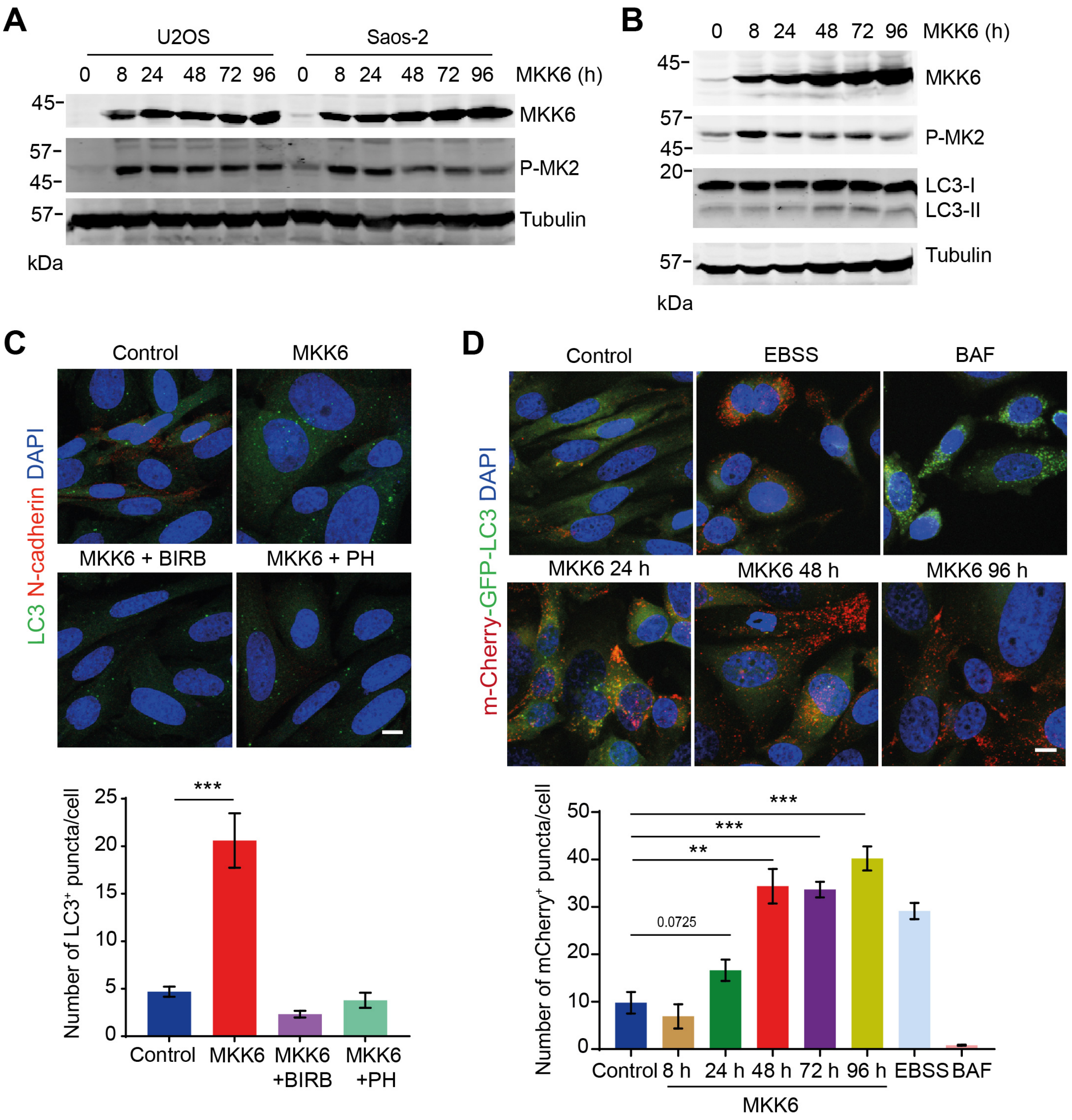

Supplementary Fig. 3. Activation of p38α suffices to induce autophagy in Saos-2 cells. Cells expressing a Tet-regulated construct were either mock treated (control) or treated with tetracycline for the indicated times to induce the expression of constitutively active MKK6. (**A**) Total lysates from U2OS or Saos-2 cells expressing MKK6 for the indicated times were analyzed by immunoblotting with the indicated antibodies. (**B**) Total lysates from Saos-2 cells expressing MKK6 for the indicated times were analyzed by immunoblotting. (**C**) Saos-2 cells expressing MKK6 for 48 h in the presence or absence of the p38α inhibitors PH797804 (PH) and BIRB796 (BIRB) were analyzed by immunofluorescence to detect $LC3^+$ puncta (autophagosomes). The histogram shows the quantification of the $LC3^+$ puncta. Bar = 10 μm. (**D**) Saos-2 cells expressing the mCherry-GFP-LC3 reporter and MKK6 for the indicated times were analyzed by immunofluorescence. As a control, cells were starved in EBSS media for 8 h, which induced autophagy and increased the number of red dots, or autophagy was inhibited with bafilomycin A1 (BAF, 200 μM) for 4 h, which decreased the number of red dots. The histogram shows the quantification of the autophagic flux as determined by

subtracting the number of green puncta from red puncta. Bar = 10 μm. Differences between control and MKK6 expressing cells were analyzed using the unpaired Student t test, (***) $p < 0.0001$, (**) $p < 0.001$.

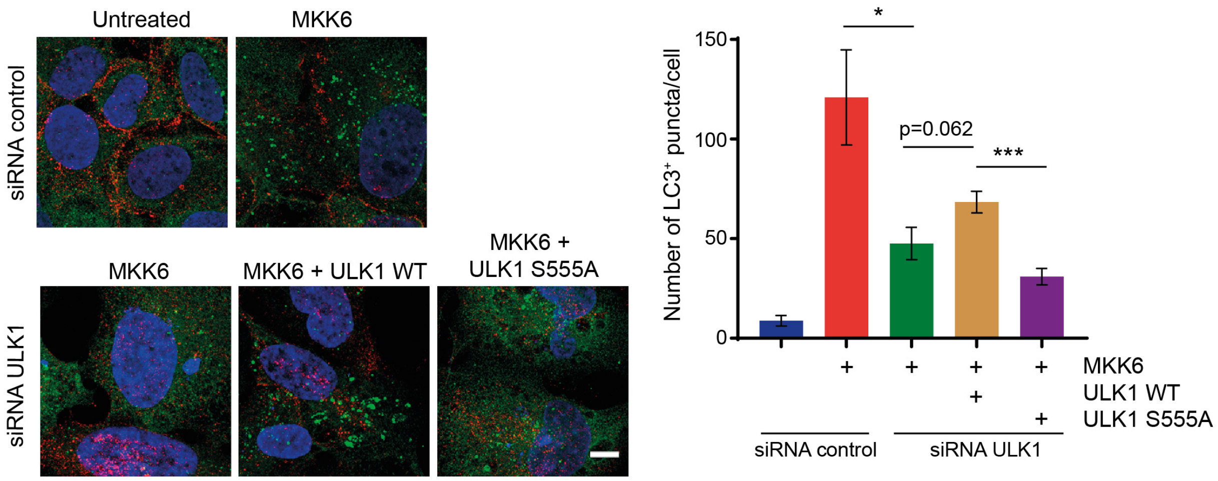

Supplementary Fig. 4. MKK6-induced autophagosome formation is impaired in cells expressing the ULK1-S555A mutant. U2OS cells expressing a Tet-regulated construct were first treated with ULK1 siRNA and 48 h later were transfected with plasmids expressing wild type (WT) or the mutant form S555A of ULK1. After 24 h, cells were either mock treated (control) or treated with tetracycline for 48 h to induce the expression of constitutively active MKK6. $LC3^{+}$ puncta (autophagosomes) were detected by immunofluorescence using LC3 antibodies. The histogram shows the quantification. Bar = 10 µm. Differences between control and MKK6 expressing cells, or between the two groups indicated were analyzed using the unpaired Student t test, (***) $p < 0.0001$, (*) $p < 0.01$.

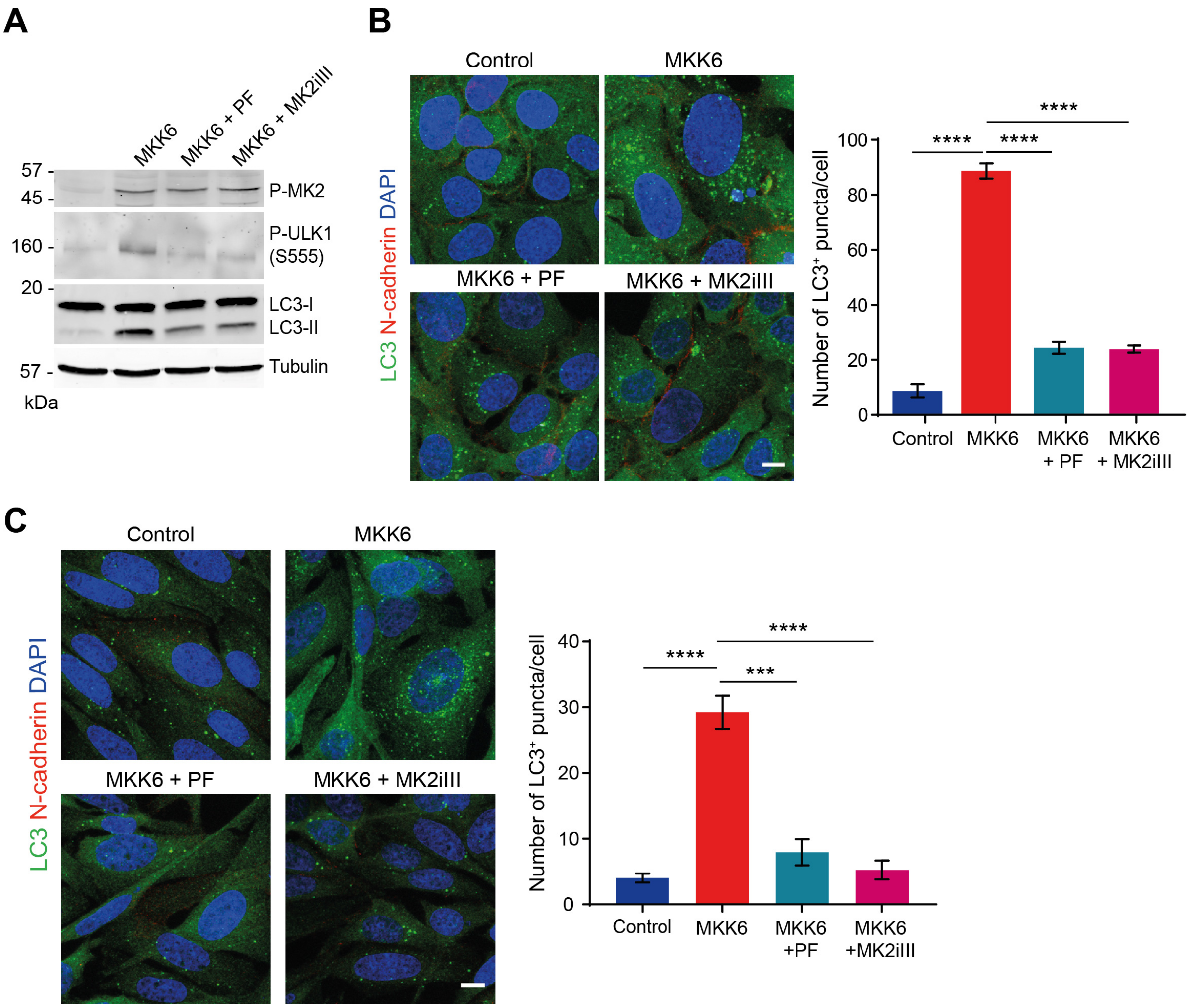

Supplementary Fig. 5. Inhibition of MK2 impairs autophagosome formation induced by MKK6. Cells expressing a Tet-regulated construct were either mock treated (control) or treated with tetracycline for 48 h to induce the expression of constitutively active MKK6 in the presence or absence of 10 µM of the MK2 inhibitors PF3644022 (PF) or MK2iIII. (**A**) Total lysates from U2OS cells were analyzed by immunoblotting using the indicated antibodies. (**B**) U2OS cells were analyzed by immunofluorescence to detect LC3$^+$ puncta (autophagosomes). Bar = 10 µm. The histogram shows the quantification of LC3$^+$ puncta. (**C**) Saos-2 cells were analyzed by immunofluorescence to detect LC3$^+$ puncta. Bar = 10 µm. The histogram shows the quantification of LC3$^+$ puncta. Differences between control and MKK6 expressing cells, or between cells expressing MKK6 in the presence or absence of MK2 inhibitors were analyzed using the unpaired Student t test, (****) $p < 0.00001$, (***) $p < 0.0001$.

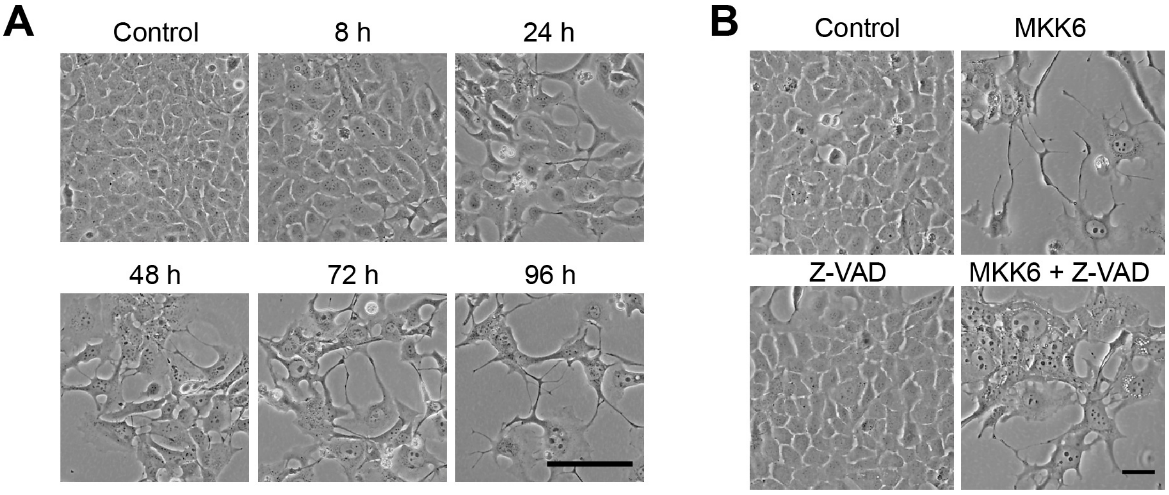

Supplementary Fig. 6. Morphological changes induced by MKK6 expression are not affected by caspase inhibition. U2OS cells expressing a Tet-regulated construct were either mock treated (control) or treated with tetracycline for the indicated times to induce the expression of constitutively active MKK6. (**A**) Changes in cell morphology upon MKK6 induction for the indicated times. Bar = 125 μm. (**B**) Morphology of cells expressing MKK6 for 96 h with or without the pan-caspase inhibitor Z-VAD. Bar = 125 μm.

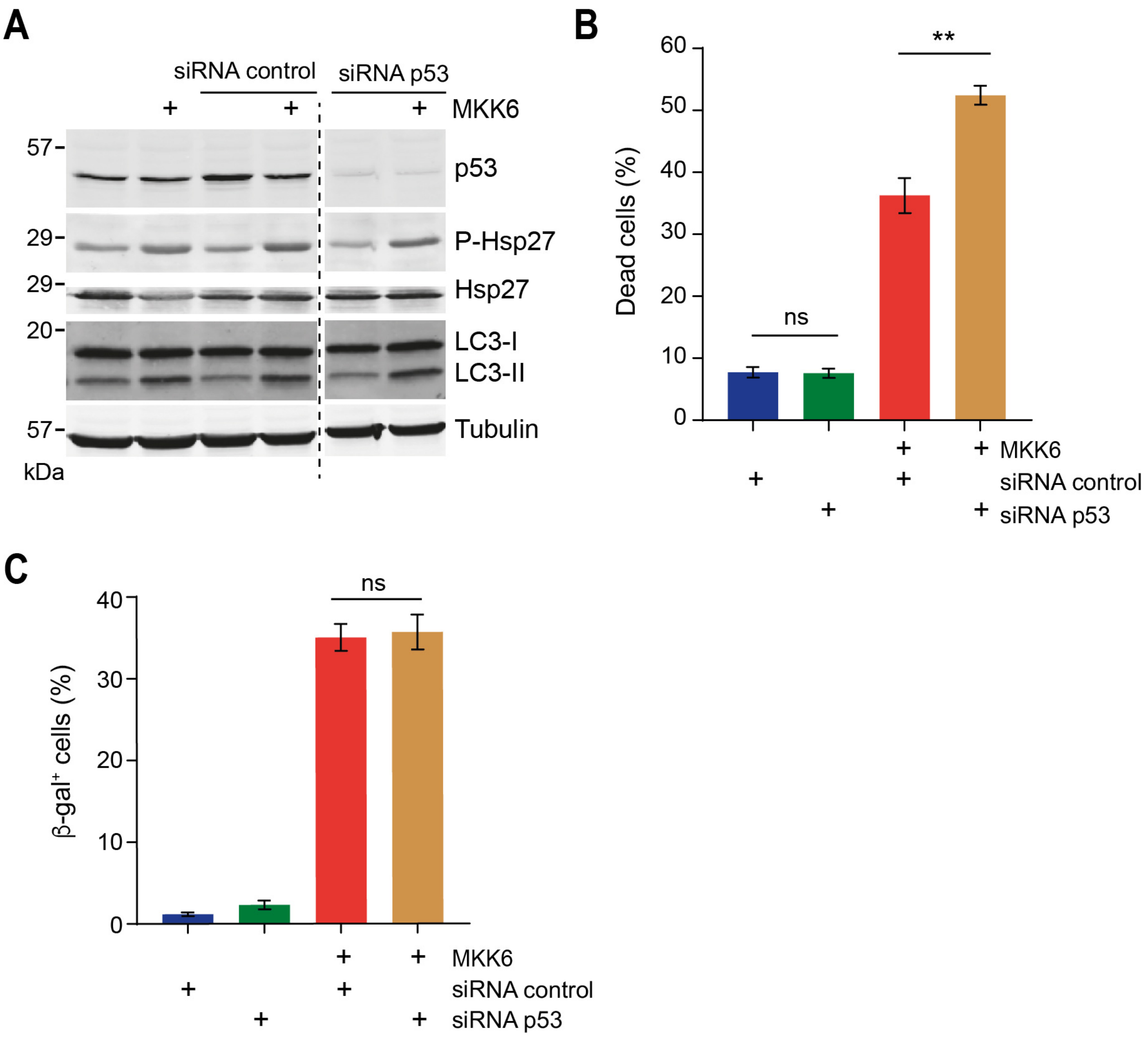

Supplementary Fig. 7. MKK6-induced autophagy and senescence do not require p53. (**A**) U2OS cells expressing a Tet-regulated construct were treated either with control (mock) or p53 siRNAs and 48 h later were treated with tetracycline for 48 h to induce the expression of constitutively active MKK6. Cell lysates were analyzed by immunoblotting using the indicated antibodies. (**B**) Cells treated as in (**A**) were analyzed for cell death as determined by FACS using Annexin V/PI staining. (**C**) Cells treated as in (**A**) were analyzed for senescence by β-gal staining. Differences between MKK6-expressing cells treated with control or p53 siRNAs were analyzed using the unpaired Student t test, (**) $p < 0.001$; ns, not significant.

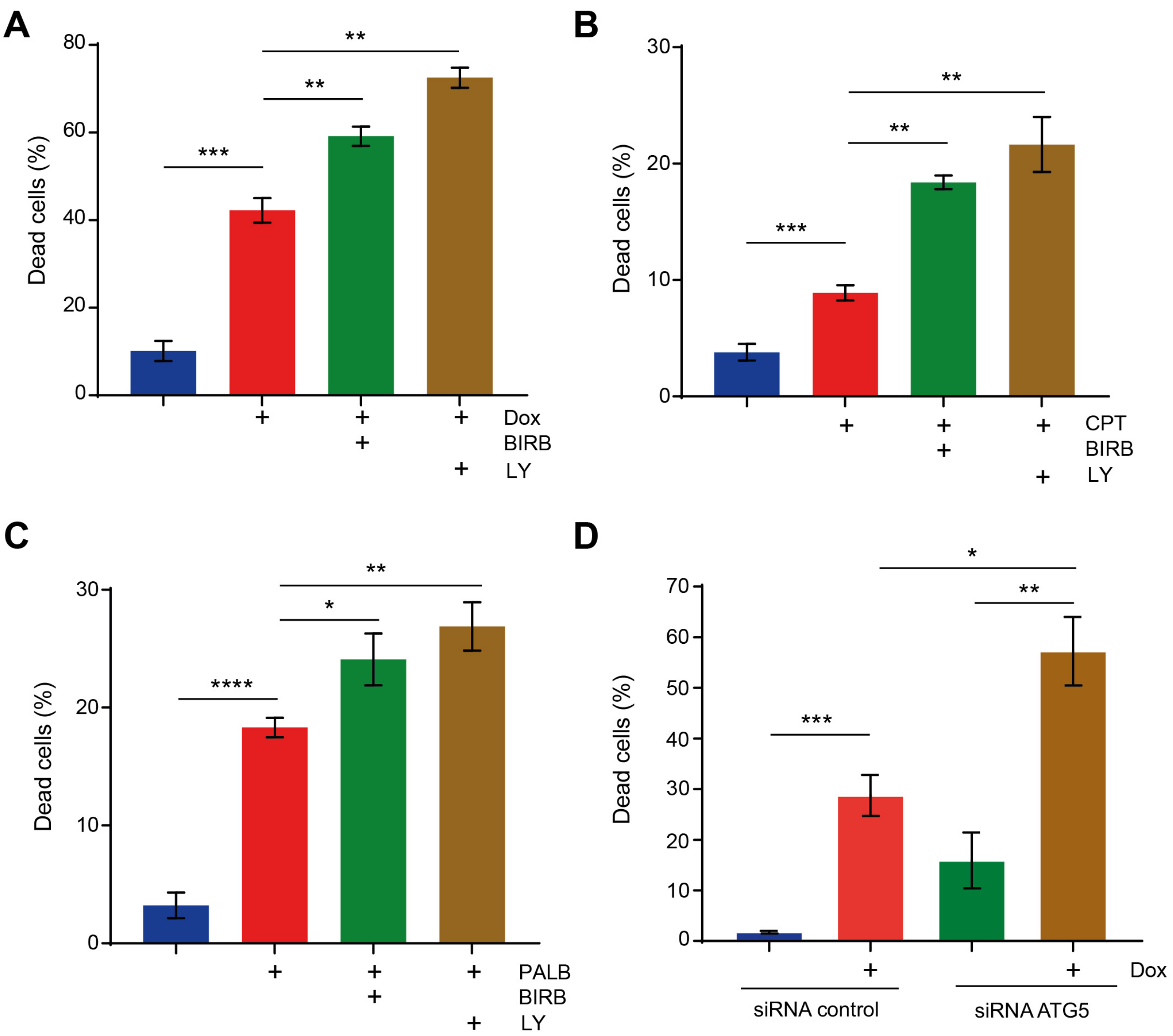

Supplementary Fig. 8. Inhibition of p38α activity sensitizes cancer cells to apoptosis. (**A**) A549 cells were treated with doxorubicin (Dox, 250 nM) for 48 h in the presence or absence of the p38α inhibitors BIRB-796 (BIRB) and LY2228820 (LY), and then were analyzed by FACS using Annexin V/PI staining. (**B** and **C**) U2OS cells were treated with camptothecin (CPT, 500 nM) (**B**) or palbociclib (PALB, 15 μM) (**C**) for 48 h in the presence or absence of the p38α inhibitors BIRB and LY, and then were analyzed by FACS using Annexin V/PI staining to determine cell death. (**D**) U2OS cells were treated with control or ATG5 siRNA and 48 h later were treated with Dox. After 48 h cell death was determined by FACS using Annexin V/PI staining. Differences between two groups of cells subjected to various treatments were analyzed using the unpaired Student t test, (****) $p < 0.00001$, (***) $p < 0.0001$, (**) $p < 0.001$, (*) $p < 0.01$.

Supplementary Table 1. Primers for RT-PCR

Primer	Sequence
CXCL8_F	ACCACCGGAAGGAACCATCT
CXCL8_R	AAAACTGCACCTTCACACAGAG
GAPDH_F	GTTTTTCTAGACGGCAGGTCA
GAPDH_R	AACATCATCCCTGCCTCTACT
IL1B_F	ATGATGGCTTATTACAGTGGCAA
IL1B_R	GTCGGAGATTCGTAGCTGGA
IL24_F	CCAGCCCTCAAGCATCACTTA
IL24_R	CAGAAGGGTCTGGCTAAAGTC
p21CIP_F	TGTCCGTCAGAACCCATGC
p21CIP_R	AAAGTCGAAGTTCCATCGCTC
SQSTM1_F	CTGCCCAGACTACGACTTGTGT
SQSTM1_R	TCAACTTCAATGCCCAGAGG

Supplementary Table 2. CRISPR guides

Gene	Transcript ID	Exon number	Guide sequence
MAPK12	ENST00000215659.12	2	TCGGCCGTGGACGGCCGCAC
MAPK14	ENST00000229795.7	2	AAGTAACCGCAGTTCTCTGT

Supplementary Table 3. Primers for sequencing

Sequence	Target plasmid
5´ - GAGGGCCTATTTCCCATGATT - 3´	Sequence of cloned oligos in pX330-EGFP
5´- ATTTAGGTGACACTATAG -3´	Sequence of mutation T180A in myc-hULK1
5´ - CCACCCAGTTCCAAACACCT -3´	Sequence of mutation S555A in myc-hULK1
